# Co-Occurrence of Familial Non-Medullary Thyroid Cancer (FNMTC) and Hereditary Non-Polyposis Colorectal Cancer (HNPCC) Associated Tumors—A Cohort Study

**DOI:** 10.3389/fendo.2021.653401

**Published:** 2021-07-13

**Authors:** Kshama Aswath, James Welch, Sriram Gubbi, Padmasree Veeraraghavan, Shirisha Avadhanula, Sudheer Kumar Gara, Esra Dikoglu, Maria Merino, Mark Raffeld, Liqiang Xi, Electron Kebebew, Joanna Klubo-Gwiezdzinska

**Affiliations:** ^1^ Metabolic Diseases Branch, National Institute of Diabetes and Digestive and Kidney Diseases, National Institutes of Health, Bethesda, MD, United States; ^2^ Department of Endocrinology, Diabetes and Metabolism, Cleveland Clinic, Cleveland, OH, United States; ^3^ National Cancer Institute, National Institutes of Health, Bethesda, MD, United States; ^4^ Department of Surgery, Stanford University, Stanford, CA, United States

**Keywords:** thyroid cancer, exome sequencing, hereditary non-polyposis colorectal cancer, familial non-medullary thyroid carcinoma, mismatch repair genes

## Abstract

Familial non-medullary thyroid cancer (FNMTC) is a form of endocrine malignancy exhibiting an autosomal dominant mode of inheritance with largely unknown germline molecular mechanism. Hereditary nonpolyposis colorectal cancer syndrome (HNPCC) is another hereditary autosomal dominant cancer syndrome which, if proven to be caused by germline mutations in mismatch repair genes (MMR)—*MLHL*, *MSH2*, *MSH6*, *PMS2*, and *EPCAM*—is called Lynch syndrome (LS). LS results in hereditary predisposition to a number of cancers, especially colorectal and endometrial cancers. Tumors in LS are characterized by microsatellite instability (MSI) and/or loss of MMR protein expression in immunohistochemistry (IHC). MSI is a rare event in thyroid cancer (TC), although it is known to occur in up to 2.5% of sporadic follicular TC cases. There are limited data on the role of germline MMR variants FNMTC. The goal of this study was to analyze the potential clinical and molecular association between HNPCC and FNMTC. We performed a cohort study analyzing the demographic, clinical, and pathologic data of 43 kindreds encompassing 383 participants (104 affected, 279 unaffected), aged 43.5 [7-99] years with FNMTC, and performed high-throughput whole-exome sequencing (WES) of peripheral blood DNA samples of selected 168 participants (54 affected by FNMTC and 114 unaffected). Total affected by thyroid cancer members per family ranged between 2 and 9 patients. FNMTC was more prevalent in women (68.3%) and characterized by a median tumor size of 1.0 [0.2-5.0] cm, multifocal growth in 44%, and gross extrathyroidal extension in 11.3%. Central neck lymph node metastases were found in 40.3% of patients at presentation, 12.9% presented with lateral neck lymph node metastases, and none had distant metastases. Family history screening revealed one Caucasian family meeting the clinical criteria for FNMTC and HNPCC, with five members affected by FNMTC and at least eight individuals reportedly unaffected by HNPCC-associated tumors. In addition, two family members were affected by melanoma. Genome Analysis Tool Kit (GATK) pipeline was used in variant analysis. Among 168 sequenced participants, a heterozygous missense variant in the *MSH2* gene (rs373226409; c.2120G>A; p.Cys707Tyr) was detected exclusively in FNMTC- HNPCC- kindred. In this family, the sequencing was performed in one member affected by FNMTC, HPNCC-associated tumors and melanoma, one member affected solely by HNPCC-associated tumor, and one member with FNMTC only, as well as seven unaffected family members. The variant was present in all three affected adults, and in two unaffected children of the affected member, under the age of 18 years, and was absent in non-affected adults. This variant is predicted to be damaging/pathogenic in 17/20 *in-silico* models. However, immunostaining performed on the thyroid tumor tissue of two affected by FNMTC family members revealed intact nuclear expression of *MSH2*, and microsatellite stable status in both tumors that were tested. Although the *MSH2* p.Cys707Tyr variant is rare with a minor allele frequency (MAF) of 0.00006 in Caucasians; it is more common in the South Asian population at 0.003 MAF. Therefore, the *MSH2* variant observed in this family is unlikely to be an etiologic factor of thyroid cancer and a common genetic association between FNMTC and HNPCC has not yet been identified. This is the first report known to us on the co-occurrence of FNMTC and HNPCC. The co-occurrence of FNMTC and HNPCC-associated tumors is a rare event and although presented in a single family in our large FNMTC cohort, a common genetic background between the two comorbidities could not be established.

## Introduction

Familial non-medullary thyroid cancer (FNMTC) comprises 3% to 9% of all NMTC cases and may manifest as a non-syndromic familial disease with thyroid cancer as the main cancer type or as a component of inherited cancer syndromes, also known as syndromic FNMTC (1). Adenomatous polyposis coli (APC)-associated polyposis, PTEN hamartoma tumor syndrome, Peutz-Jeghers syndrome, Carney’s complex, and DICER1 are syndromic forms of FNMTC that follow an autosomal dominant mode of inheritance, whereas Werner’s syndrome, Pendred syndrome, and ataxia telangiectasia are autosomal recessive types of syndromic FNMTCs (1). Although the molecular basis for syndromic FNMTC is largely uncovered, the genetic background of non-syndromic FNMTC is grossly undetermined. Therefore, non-syndromic FNMTC is currently diagnosed solely on clinical grounds, based on the presence of epithelial cells-derived thyroid cancer (TC) in two or more first-degree family members and exhibiting an autosomal dominant pattern of inheritance with incomplete penetrance, in the absence of any syndromic NMTC manifestations or environmental causes. There are mixed results regarding the aggressiveness of tumors in FNMTC cohorts, with some data suggesting more aggressive behavior of FNMTC, whereas others revealing similar biology to the sporadic cases (2, 3). However aggressiveness is more evident in families with three or more FNMTC affected members than in families with two affected members (4). FNMTC is two to three times more prevalent in women than in men (5). Reports on the age of diagnosis between sporadic and familial NMTC are varied (2, 5, 6). Management of FNMTC is quite challenging as there are no national or international guidelines addressing screening, surveillance, optimal procedures, and follow-up (7). One of the reasons for the lack of unequivocal screening strategy is the unknown genetic background of FNMTC. Several low penetration susceptibility risk loci or genes (i.e., TTF1, FOXE1, SRGAP1, SRRM2, HABP2, MAP2K5, and DUOX2) have been identified, but none has been uniformly shown to have a causative role (8). Over the years, studies across various FNTMC cohorts have explored genetic landscapes and described many potential susceptibility genes (9, 10), low penetrance variants (11, 12), and susceptibility loci (13), and have analyzed role of miRNA and telomere lengths across FNMTC families (14) using whole exome, whole-genome sequencing, qPCR, and linkage analysis techniques, with limited success in identifying a strong candidate gene for FNMTC. Recently established role of the codon bias in TC-related genes, including non-synonymous mutations, may add to the understanding of the molecular mechanisms involved in TC and its familial forms (15). To date, there is no genotype-phenotype correlation in FNMTC that could guide screening strategy and genetic counselling.

The hereditary non-polyposis colorectal cancer (HNPCC) is also an autosomal dominant disease that is clinically diagnosed based on the Amsterdam II criteria of ≥3 members of the family that are affected across at least two generations with at least one member of the family presenting with HNPCC-associated tumor below the age of 50 years, and familial adenomatous polyposis (FAP) is ruled out in any colorectal cancer (CRC) cases (16). Although HNPCC is a clinical diagnosis, the Lynch syndrome (LS) refers to HNPCC caused by germline mutation in mismatch repair genes (MMR) (17). HNPCC tumor spectrum includes colon, small bowel, endometrial, stomach, ovarian, pancreas, uterus, renal, biliary tract and brain tumors, sebaceous gland adenomas, and keratoacanthomas of the skin or any solid tumor characterized by microsatellite instability (MSI). High penetrant pathogenic germline mutations in at least one of the mismatch repair (MMR) genes—*MLHL*, *MSH2*, *MSH6*, *PMS2*, and *EPCAM*—predispose the LS tumors to a high frequency of replication errors leading to microsatellite instability (MSI) (18). MSI is a rare event in thyroid cancer; however, it has been observed in about 2.5% of patients with sporadic follicular TC (19). The role of MSI due to germline pathogenic variants in MMR genes in FNMTC and the frequency of the association of FNMTC and HNPCC co-morbidities is unknown.

## Methods

### Study Design

We performed a prospective cohort study at the National Institutes of Health (NIH) Clinical Center between April 2010 and June 2020 (Clinicaltrials.gov: NCT-01109420). Written informed consent was obtained from all participants. The study was approved by the NIH Institutional Review Board. The main inclusion criteria for the study were the presence of at least two first-degree relatives affected with NMTC and older than 7 years. Individuals with any known syndromic FNMTC were excluded from the study. Demographic, clinical, and pathologic data were collected from medical records, family history questionnaires, and patient interviews. Patient histories, family pedigrees and physical examinations, imaging studies, and laboratory tests were obtained from all patients enrolled in the protocol. All pathology slides were reviewed at the authors’ institution to confirm the TC diagnoses and histologic subtype.

### Whole-Exome Sequencing, Variant Calling, and Evaluation

Peripheral blood DNA was extracted from the subset of FNMTC cohort and sequencing libraries were made using Agilent Sure Select human all exon V5 + UTR capture kit enabling targeted sequencing of the exons and sequenced as PE 101 on Illumina HiSeq at a mean coverage of ~76×, yielding ~95% mapped reads across a total of ~70M reads, as described previously (9).

Raw FASTQ files from sequencing the FNMTC subset, were adaptor trimmed using Cutadapt (v2.9), reads were mapped to the human genome build hg38, using Burrows Wheeler Alignment tool—maximal exact match (BWA-MEM) v0.7.17 (20). Base quality score recalibration (21), single nucleotide polymorphism (SNP) genotyping, and genotype level refinement was computationally implemented according to Genome Alignment Tool Kit (GATK) v4.1.8.0, best practices for germline short variant analysis (22, 23). Impact of the filtered variants across the MMR genes and their clinical significance was evaluated *via* variant effect predictor (VEP) from Ensembl genome browser (24), clinical genomic variation database ClinVar (25), and COSMIC database (26). The effect of amino acid change because of missense mutation on the protein was modeled using Missense3D from Expasy (27). Population prevalence statistics were obtained using the genome aggregation database (gnomAD) (28). *In silico* modeling outcomes on pathogenicity were extracted from VEP and VarSome (29). Extensive literature search on the variant of interest was performed using PubMed.

### Confirmatory Multi-Panel Targeted Sequencing

Confirmatory targeted sequencing was performed on the proband’s saliva by Invitae across multi-cancer, breast and gynecological cancer, thyroid cancer, renal/urinary tract, and melanoma panels, including the available add-on preliminary-evidence genes across each of the panels. Genomic DNA from this sample was enriched for targeted regions using a hybridization protocol and sequenced at ≥50× depth. Bioinformatics analysis of the targeted sequencing data was performed by Invitae using their proprietary pipeline, and the sequence changes identified were interpreted in a clinical context of single clinical transcript and analyzed using the Sherloc algorithm (30).

### Immunohistochemical (IHC) Staining of Available Tumor Tissue

IHC was performed on FFPE tissue taken from representative sections (4 µm thick) of the available TC samples to evaluate the expression of the MMR proteins *MLH1, MSH2, MSH6, PMS2* as per the manufacturer’s protocol (Roche Laboratories) and performed on Ventana Benchmark Ultra Auto stainers. Proficient mismatch repair (pMMR) or deficient mismatch repair (dMMR) status was assessed by two independent pathologists (MM, ED) based on H&E and IHC staining pattern. H&E and immunohistochemical images were taken using a Leica Camera.

### MSI Analysis and TSO500 Gene Panel Sequencing of the Available Tumor Tissue

DNA and RNA were extracted from formalin-fixed paraffin embedded available TC tissue samples obtained at surgery using QIAmp DNA FFPE/RNeasy FFPE kit (Qiagen). Capture-based next-generation sequencing was performed using the TruSight Oncology 500 Gene Panel, a Clinical Laboratory Improvement Amendments (CLIA)-validated commercial pan-cancer panel with DNA+RNA assay targeting 523 genes designed to detect single-nucleotide variants (SNV), insertions and deletions (INDEL), copy number variants (CNV), and gene fusions across 523 cancer genes, including major driver genes in thyroid oncogenesis, evaluating tumor mutation burden (TMB) and MSI. Proprietary TruSight Oncology 500 v2.1 local application from Illumina was used in bioinformatics analysis and in determination of TMB and MSI. Classification of variants and actionability of variants was determined by algorithms developed by QCI (QCI interpret) that is based on multiple clinical and population-based bioinformatics databases and experimental and clinical data reported in biomedical journals.

### *BRAFV600E* Mutation Analysis from Available Cytology Samples of Thyroid Cancer

DNA was extracted from available cytology samples obtained at fine-needle aspiration biopsy of the thyroid nodule and subjected to mutant allele enriching, Competitive Allele-Specific TaqMan^®^ PCR (castPCR™) on a ViiA7 real-time PCR system (Applied Biosystems), using a single primer set encompassing codon V600E of the *BRAF* gene, for detection of the V600E (c.1799T>A) mutation. CastPCR combines *BRAF*-specific TaqMan^®^ qPCR with *BRAF*-WT-specific minor groove binder (MGB) blockers to suppress non-specific amplification of the wild type allele.

### Statistical Analysis

Data were summarized by using frequencies and percentages for categorical variables and means with standard deviations (SDs) or medians with the range [min-max] for continuous variables, dependent on the distribution. Shapiro–Wilk’s normality test was performed to access normal distribution. Data analysis was performed using GraphPad Prism version 9.

## Results

### Clinical Analysis of FNMTC Cohort

**Figure 1:**
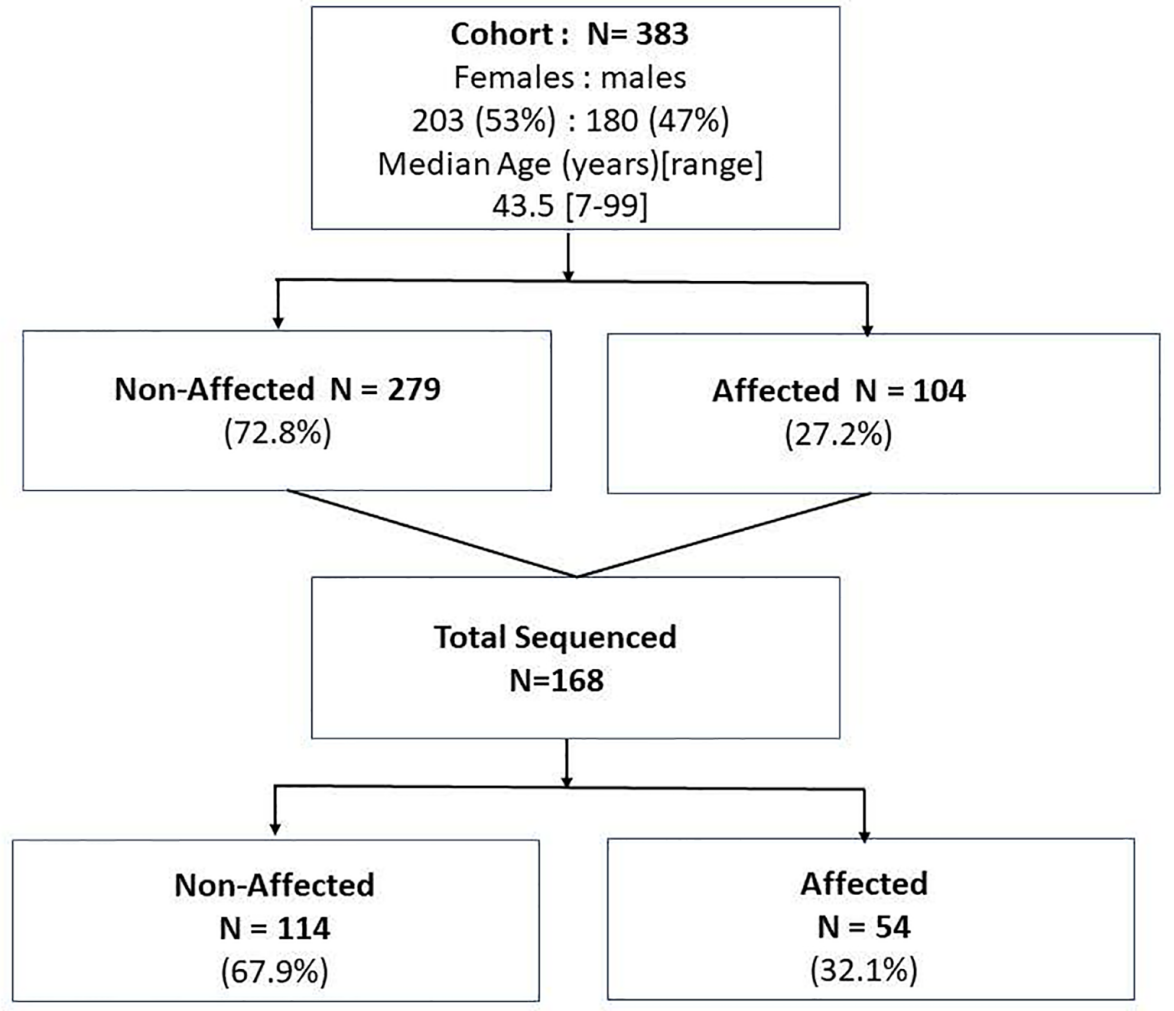
The study population of the affected and non-affected by FNMTC participants that underwent clinical evaluation and subjects who in addition to clinical evaluation underwent whole exome sequencing of the blood/saliva samples.

Forty-three FNMTC families encompassing 383 family members that met the inclusion criteria for FNMTC (104 affected, 279 unaffected), mean age 43.5 [7-99] years, were selected for the study. A total of 168 members were sequenced, encompassing 54 FNMTC affected and 114 unaffected members from the cohort (**Figure 1**). Total affected by TC members per family ranged between 2 and 9 patients. FNMTC was more prevalent in women (68.3%) and characterized by a median tumor size of 1.0 [0.2-5.0] cm, multifocal growth in 44%, and gross extrathyroidal extension in 11.3% (**Table 1**). Central neck lymph node metastases were found in 40.3% of patients at presentation, 12.9% presented with lateral neck lymph node metastases, and none had distant metastases. Papillary thyroid cancer (PTC) presented as the predominant cancer type in the cohort (**Table 1**). Because there are reports on more aggressive behavior of familial micro-PTC (31), we analyzed the clinical features of micro-PTC in our study. Within the studied FNMTC cohort, 24 patients presented with micro-PTC. Ten of 24 patients with micro-PTC (41.7%) presented with multifocal disease, three (12.5%) patients had central neck lymph node metastases, and one (4.2%) patient presented with lateral neck lymph node metastases. There was no microscopic or gross extra-thyroid or extra-nodal extension of micro PTC in the FNMTC cohort. A typical, less aggressive behavior of micro-PTC in our cohort, could have been due to implementing an active screening strategy.

**Table 1:** Clinical characteristics of the study participants affected by thyroid cancer.

Clinical and pathologic features of patients affected by thyroid cancer (N=104)	
Median Age (years) [range]	40 [17-76]
Gender	Totals
Female (%)	71/104 (68.3%)
Male (%)	33/104 (31.7%)
Histology	Totals
PTC (%)	95/104 (91.3%)
PTCFV (%)	7/104 (6.7%)
PTCTC (%)	2/104 (2%)
Median tumor size (cm) [range]	1 [0.2-5]
Multifocal growth*	26/59 (44%)
Gross extra thyroid extension^	7/62 (11.3%)
Lymph node metastastases**	
Central neck	25/62 (40.3%)
Lateral neck	8/62 (12.9%)
Median number of positive lymph nodes [range]	5 [1-13]
Median number of lymph nodes examined [range]	5 [1-35]
Distant metastases	0/104 [0%]

PTC, papillary thyroid cancer; PTCFV, follicular variant of papillary thyroid cancer; PTCTC, tall cell variant of papillary thyroid cancer.

*Data on tumor multifocality available for 59 patients.

^Data on extrathyroidal extension available in 62 patients.

**Data on presence or absence of lymph node metastases available in 62 patients.

### Description of a Kindred With HNPCC and FNMTC

**Figure 2:**
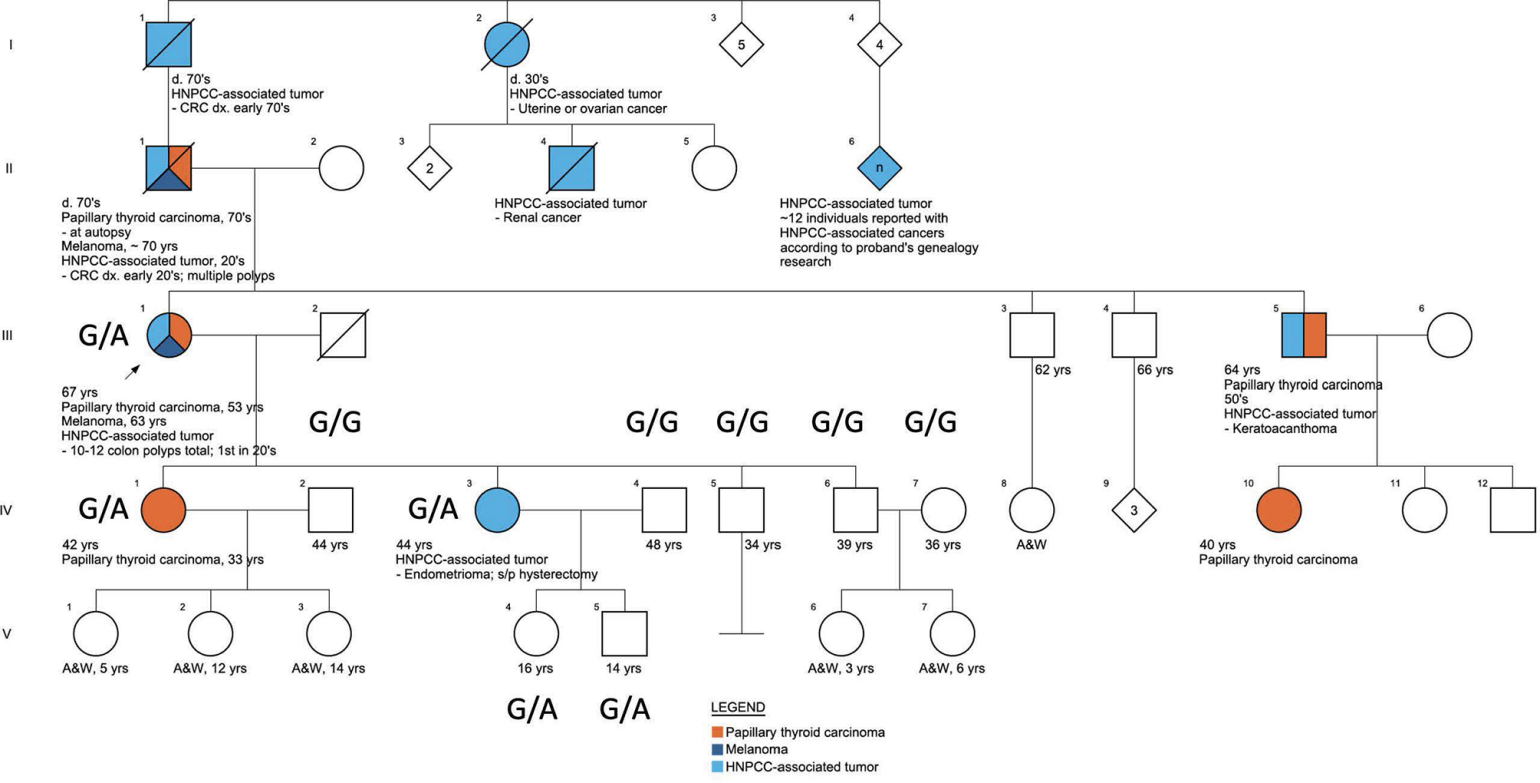
The pedigree of the family characterized by the presence of FNMTC-HNPCC, associated tumors, and melanoma.

Screening for comorbidities based on patients’ questionnaires (**Supplementary PDF 1**) and clinical evaluation revealed that one of the enrolled Caucasian families met the clinical Amsterdam II criteria for HNPCC diagnosis, based on the following family medical history reported by the proband (III.1), **Figure 2**. Eight family members presented with HNPCC-associated tumors: three had colorectal tumors (I.1 with CRC in his 70s, II.1 with CRC in his early 20s, and III.1 with multiple colon polyps during her lifetime with the first removed in her 20s). Two family members had ovarian or endometrial tumors (I.2 and IV.3). One family member reportedly had a keratoacanthoma (III.5) and another had kidney cancer (II.4). Additionally, the proband, who was the pedigree informant, reported that her research into the family genealogy discovered up to a dozen family members on her affected father’s side of the family with unspecified Lynch-like tumors (II.6). HNPCC-associated tumors were present in several members before the age of 50 years, and non-typical tumors, such as melanoma, were observed in two family members (II.1 and III.1). From the perspective of the proband’s deceased affected father (II.1), this family fulfills the Amsterdam II criteria based on the reported manifestations and has a PREMM_5_ score of 6.7% to 6.9% (32), thus meeting the criteria for genetic testing for germline MMR mutation, given that PREMM_5_ score over 2.5%% to 5% is recommended for LS genetic evaluation. Unfortunately, because this person with the youngest age of CRC onset was deceased, the proband (III.1) was the closest relative for whom genetic evaluation could take place. Although the proband (III.1) did not develop CRC, this could have been skewed by the early age at which she started colonoscopies because of her father’s early age of CRC diagnosis, and those colonoscopies did start finding and removing polyps in her 20s. The proband (III.1) had FAP excluded by documented normal sequencing and deletion/duplication testing of the gene APC in a CLIA lab. PTC was diagnosed in five family members (II.1, III.1, III.5, IV.1, and IV.10) across three generations (**Figure 2**).

### Genetic Characterization and Evaluation of the Variant of Interest in the Kindred

Across the FNMTC cohort who underwent sequencing (N=168), we identified in total 2931 variants across all eight MMR genes (*MSH2, MLH1, MSH6, PMS2, MLH3, MSH3, PMS1*, and *EPCAM*; **Supplementary Table 1**). A heterozygous missense variant (rs373226409 (G>A)) was detected in Chr2:47476481 within the *MSH2* gene exclusively in one kindred exhibiting manifestations of both FNTMC and HNPCC-associated tumors. In this Caucasian family, the sequencing was performed in one member affected by FNMTC, HPNCC-associated tumors and melanoma (III.1), one member affected solely by HNPCC-associated tumor (IV.3), and one member with FNMTC only (IV.1), as well as seven unaffected family members (IV2, IV4-7, V.4, V5). This variant—a missense mutation (c.2120G>A; p.Cys707Tyr)—was present in this family in three affected adults (III.1, IV.1, and IV.3), and in two unaffected children under the age of 18 (V.4, V.5) and was presenting as G/G homozygous in non-affected adults (IV.2, IV.4, IV.5, IV.6, IV.7) (**Figure 2**) from this family and across the rest of the cohort (**Supplementary Figure 1**). The presence of this variant was confirmed by targeted sequencing performed on the proband’s sample using multiple cancer panels across 103 genes at a CLIA-certified genetics laboratory. We did not find any other genetic alterations in the kindred.

Missense3D from Expasy was used to model the effect of the predicted amino acid change Cys707Tyr, which revealed a switch with a change between buried cysteine (relative solvent accessibility (RSA) of 3.7% and exposed state of the target variant tyrosine (RSA 16.2%) residue (**Supplementary Figure 2**), predicting structural damage (27). Screening of the cancer somatic mutations database COSMIC, for rs373226409 (G>A) variant in *MSH2* identified a single hit with a somatic mutation ID COSV51880288, a male tumor sample P-0003841-T01-IM5 with bladder urothelial carcinoma and an unknown status of MSI, with allele frequency of 0.66 and a FATHMM *in-silico* model prediction of pathogenicity (score, 0.99) (33). *In silico* models SIFT and PolyPhen from VEP (24) predicted a deleterious (0) and probably damaging (0.961) outcome, respectively, for our variant of interest, with mixed clinical significance. An *in-silico* analysis of *MLH1, MSH6*, and *MSH2* variants in a Pakistani cohort using seven *in silico* models reported this *MSH2* variant as pathogenic across five of the seven *in silico* models (34) and currently 17 of 20 *in silico* models in VarSome (29) predict deleterious effect of this variant. ClinVar, a database of genomic variations and human health, presented mixed outcomes of pathogenicity across various CLIA laboratory submissions evaluated post 2014 and cite this variant as of uncertain significance and/or likely benign. A low prevalence of this variant with a minor allelic frequency (MAF) of 0.00006 in Caucasians and a relatively high prevalence in the South Asian population with a MAF of 0.003 was indicated by gnomAD. The relatively high prevalence in the South Asian population was used by the Invitae variant classification algorithm Sherloc to categorize this variant as likely benign.

### Germline Variant in *MSH2* (c.2120G>A) Is Not Associated With *MSH2* Loss of Nuclear Expression in Available TC Tissues

**Figure 3:**
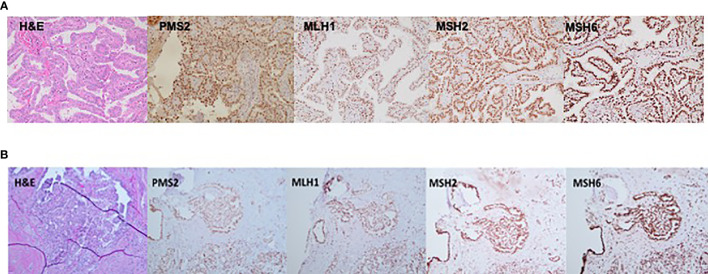
Immunostaining (20×) demonstrating an intact nuclear expression of mismatch repair (MMR) proteins—*PMS2, MLH1, MSH2*, and *MSH6* in thyroid cancer tissue derived from **(A)** patient III.1 and **(B)** patient IV.1.

TC tissue samples were available for analysis for two family members, III.1 and IV.1. Patient III.1 had a multifocal classic papillary thyroid cancer affecting both thyroid lobes (largest focus, 2 cm), with capsular invasion and extrathyroidal extension and bilateral central and left lateral neck lymph node metastases in 12/26 lymph nodes examined, consistent with AJCC staging T4N1bM0. Patient IV.1 was characterized by a classic micro-papillary thyroid cancer measuring 0.4 cm × 0.4 cm × 0.3 cm without lymph node metastases (zero of one lymph node examined), consistent with T1aN0M0 AJCC stage. IHC staining for *MSH2, MSH6, MLH1*, and *PMS2* revealed intact nuclear expression of all markers in both tumors (**Figures 3A, B** **)**.

### Molecular Testing of Available Thyroid Tumor(s) Revealed Somatic *BRAF*V600E Mutation and Microsatellite Stable Status

The DNA extracted from tumor IV.1 (papillary microcarcinoma) was insufficient to perform TruSight Oncology 500 gene panel sequencing. However, castPCR assay detected a *BRAF*(c.1799T>A;p.V600E) mutation in this tumor. TruSight Oncology 500 analysis of the proband’s (III.1) tumor revealed an MSI-stable status with a tumor mutation burden (TMB) of 1.57 mutations/megabase. TruSight Oncology gene panel V2 also detected *BRAFV600E*, a somatic pathogenic mutation in the proband’s tumor along with additional somatic VUSs listed in **Supplementary Table 2**. The variant of interest in *MSH2* (c.2120G>A) was detected by the targeted sequencing and categorized as a variant of uncertain significance (VUS) with a variant allele frequency (VAF) of 49%, consistent with its germline presence and inconsistent with somatic loss of heterozygosity at that locus. No CNVs or gene fusions or rearrangements were reported.

## Discussion

HNPCC and FNMTC are both autosomal dominant diseases and are known to present with a strong familial predisposition. Clinically, FNMTC presents with epithelial-derived TC as the index cancer, while the HNPCC tumor spectrum is variable and, apart from classic colorectal and endometrial cancers, could include a spectrum of extra-colonic tumors of the brain, ovary, hepatobiliary tract, pancreas, sebaceous skin glands (including keratocanthoma), stomach, and ureter/renal pelvis (35). Notably, benign colon polyps have also been associated with HNPCC (36, 37), a phenomenon also observed in the index FNMTC-HNPCC kindred. The frequency of LS is estimated at 0.05% to 0.3% in the general population (38) and varies across different cancer populations, up to 5.8% (39). Data on prevalence of LS among patients with CRC from various cohorts across different ethnic populations are quite variable. A cumulative study across six hospitals from the Ohio metropolitan area noted an overall prevalence of LS across their cohort with colorectal cancer (N = 1,566) at 2.8% (40, 41), which was similar to a Finnish population study across nine hospitals that estimated a prevalence of LS among patients with CRCs (N = 1,044) as 2.7% and an Icelandic CRC cohort (N = 1182) with estimated prevalence of 2.5% (42, 43). On the other hand, an Australian study (N = 509) estimated LS prevalence in their CRC cohort to be as twice as high at 5.2% (44), whereas a Spanish EPICOLON multicenter nationwide study (N=2,093) and a Japanese hospital based study (N=1,234) projected a prevalence of LS in their CRC cohorts at significantly lower rates of 0.7% (45, 46). Data on the prevalence of LS in patients with endometrial cancers are still evolving. A 2013 study across Spanish patients (N = 173) with endometrial cancer documented a prevalence of 4.6%, which was six-fold higher than the prevalence of LS among Spanish patients with colorectal cancer (0.7%) as reported in a 2012 study (47). Studies from screening the North American population of patients with endometrial cancer estimated a prevalence of LS between 1.8% and 4.5%. The variation in prevalence estimates of LS across various studies could be due to insufficient data accumulation, the effect of environmental factors, or ethnic predispositions of these cohorts. We present a 2.1% prevalence of HNPCC across our FNMTC cohort. This, to our knowledge, is the first report of co-occurrence of these two comorbidities. HNPCC is a clinical diagnosis, which, if attributed to germline pathogenic variants in MMR genes, could be diagnosed as LS. However, up to 50% of the families that meet the Amsterdam II criteria, have no known heritable MMR defects (48, 49)

The underlying cause for LS is attributed to defects in MMR genes leading to MSI in the tumors, which is associated with higher likelihood of pathogenic mutations and clonal growth (50). On the contrary, the genetic basis for FNMTC susceptibility remains largely unknown (7–12), and FNMTC is not a typical part of the known LS spectrum of tumors. However, previous studies have presented TC as a rare LS-associated cancer in carriers of germline MMR gene mutations (51) and a handful of case reports document ATC and undifferentiated thyroid cancers (35, 52) and PTC in LS patients (53, 54), either with or without MSI in the analyzed tumors. However, none of the abovementioned studies have presented FNMTC as a part of the LS comorbidity.

A few studies have investigated the role of germline MMR mutations and susceptibility to certain subtypes of TC using a combination of IHC, MSI, and/or next-generation sequence analysis in the tumor tissue. A study on a Portuguese population aimed at evaluating a panel of germline MMR SNPs suggested involvement of a specific *MSH6* SNP in NMTC susceptibility, although a large, multicentric study with independent datasets of patients is required to confirm these findings (55). Interestingly, MMR-deficient status of *MSH2* and *MSH6* has been observed in aggressive forms of TC, such as anaplastic TC (56). This observation is consistent with a recent report showing that MSI status and MMR deficiency likely plays a role in TC dedifferentiation, as documented by MMR-deficient status in de-differentiated tumors such as poorly differentiated and anaplastic TC (57). Moreover, a study looking at oxidative stress and MMR activity across follicular TC, follicular thyroid adenomas and PTC demonstrated intact expression of MMR in follicular TC and adenomas and a lower expression of MMR genes in PTC under oxidative stress (58). On the other hand, studies of the expression patterns of MMR genes in thyroid tumor tissues demonstrated significantly higher expression of *MLH1, MSH2*, and *PMS1* in malignant tumor compared to benign lesions (59). Prevalence of MSI is not well explored in TC and few studies have investigated the prevalence of MSI in TC. Le et al. report a ~2% prevalence of MSI in TC but mostly at late and advanced stages (60). A study by Genutis et al., investigating the prevalence of MSI across various subtypes of TC, reported MSI in 2.5% of follicular TCs but no evidence of MSI in PTC, anaplastic, medullary, and poorly differentiated TC (19). Similar findings were obtained in a study evaluating PTC by genomic hybridization and MSI analysis showing no MSI in any of the PTC samples (61). Although several studies have confirmed the pivotal role of MSI in tumorigenesis due to MMR mutations in LS (19, 51, 59, 62–64), the altered expression of MMR genes, its role in various subtypes of TC and its pathogenesis is yet to be well understood.

The Caucasian FNMTC-HNPCC kindred presented in this study outlines several interesting clinical and genetic observations across the affected family members. First, this family presents co-occurrences of FNMTC and melanoma that are not a usual part of HNPCC-associated tumors spectrum. Second, the missense c.2120G>A variant detected in the *MSH2* gene was present exclusively in this family and segregated as heterozygous G>A across sequenced-affected adults and in two children of one of the affected adults, below the age of 18 years. Although the children are not affected with any symptoms from either condition, CRCs are extremely rare in pediatric populations (65) and extra-colonic tumors usually are known to present in the third decade of life (66). However, careful clinical monitoring in the younger members with familial history is imperative. We lack blood or saliva samples from the other living-affected members of this family to understand better if the variant segregates as heterozygous in all affected individuals. Although 17 of 20 *in silico* models from VarSome (29) predicted a deleterious outcome for this variant and 3D modeling of the amino acid Cys707Tyr predicting structural damage, there are no high-quality pre-clinical experimental data supporting the pathogenicity of this variant and 3D Expasy prediction of buried/exposed switch in ATP-ase domain effect because this mutation needs to be further investigated. In our study, the affected members investigated were characterized by microsatellite-stable thyroid tumors with preserved nuclear expression of MMR proteins in the tumor tissue. Moreover, despite a very low prevalence of the variant of interest in the Caucasian population, its relatively high prevalence across the South Asian population (MAF of 0.003), which is not characterized by a significantly higher LS prevalence, renders *MSH2* c.2120 G>A (p.Cys707Tyr) variant unlikely to be causative. However, recent data suggest an association of this variant with HNPCC/colon cancer in the South Asian population (34). Unfortunately, the CRC tissue was not available for us to analyze potential involvement of *MSH2* c.2120 G>A (p.Cys707Tyr) in the pathogenicity of colon tumors in the analyzed kindred.

A *BRAF* V600E mutation was identified in available thyroid tumors from family members with PTC. Approximately 40% to 45% of PTCs are known to be *BRAF* V600E positive (67). Hence, the presence of this mutation in PTC profiling of the proband and the proband’s daughter, who also presented with the *MSH2* variant of interest, indicates *BRAF*V600E as the likely driver somatic oncogene in PTC, rather than the *MSH2* variant of interest. One possible explanation for the detection of protein expression *via* IHC could be that the described missense mutation in MSH2 gene, leading to Cys>Tyr change produced a truncated protein with intact antibody binding site and hence a positive staining from IHC. More functional studies are required to further investigate this phenomenon.

In summary, this study, to our knowledge, is the first to report a co-occurrence of FNTMC and HNPCC. Parallel to this finding, this family also presents with other unusual cancers that are not typical of HNPCC, warranting further investigation of potential molecular background in common between the two conditions. One of the strengths of our study is that we have performed an extensive screening of comorbidities across a large FNMTC cohort with a thorough medical questionnaire and long-term follow-up as a prospective study. The major limitation is the lack of availability of more than two thyroid tumor samples from the family or any non-thyroid tumor for MSI and MMR expression analysis.

In conclusion, this is the first report known to us on the co-occurrence of FNMTC and HNPCC. The co-occurrence of FNMTC and HNPCC-associated tumors is a rare event and although presented in a single family in our large FNMTC cohort, a common genetic background between the two comorbidities could not be established. The *MSH2* variant we observed in this family is unlikely to be an etiologic factor of FNMTC predisposition in these kindred. The reported rare co-occurrence of FNMTC and HNPCC-associated tumors and melanoma suggests a higher likelihood of a common genetic background yet to be discovered, rather than by chance occurrence as etiology.

## Data Availability Statement

The data generated in this manuscript can be found in NCBI using accession number PRJNA717713.

## Ethics Statement

The studies involving human participants were reviewed and approved by NIH institutional review board. Written informed consent to participate in this study was provided by the participants’ legal guardian/next of kin.

## Author Contributions

We thank our patients for participation in the study. We thank Drs. Lee S. Weinstein and Sunita Agarwal for a critical review of the paper. KA—conceptualization, data curation, formal computational analysis, methodology, and writing—original draft. JW—genetic counseling, clinical care, reviewing, and editing the manuscript. SG—clinical care, reviewing, and editing the manuscript. PV—clinical care, reviewing, and editing the manuscript. SA—clinical care, reviewing, and editing the manuscript. SKG—data curation, reviewing, and editing the manuscript. ED—pathology evaluation, immunostaining, reviewing, and editing the manuscript. MM—pathology evaluation, immunostaining, reviewing, and editing the manuscript. MR—molecular analysis of the tumor, reviewing, and editing the manuscript. LX—molecular analysis of the tumor, reviewing, and editing the manuscript. EK—conceptualization, methodology, reviewing, and editing the manuscript. JK-G—conceptualization, resources, data curation, supervision, clinical care, project administration, reviewing, and editing the manuscript. All authors contributed to the article and approved the submitted version.

## Funding

The study was supported by the NIH intramural funding ZIE DK04705313. 

## Conflict of Interest

The authors declare that the research was conducted in the absence of any commercial or financial relationships that could be construed as a potential conflict of interest.
