# Supplementary material for: Co-Occurrence of Familial Non-Medullary Thyroid Cancer (FNMTC) and Hereditary Non-Polyposis Colorectal Cancer (HNPCC) Associated Tumors—A Cohort Study
Source: Front Endocrinol (Lausanne). 2021 Jul 13;12:653401. doi: 10.3389/fendo.2021.653401 (PMC8315151; doi:10.3389/fendo.2021.653401)
Supplement: Supplementary file 1 [file DataSheet_1.pdf]

______________________
Family Number

# FAMILY HISTORY QUESTIONNAIRE

For each question, please check or record the appropriate response. If your answer does not fit one of the responses provided, feel free to write in your answer.

STATEMENTS IN ALL CAPITAL LETTERS ARE INSTRUCTIONS TO YOU. SUCH STATEMENTS MAY INSTRUCT YOU TO SKIP CERTAIN QUESTIONS OR THEY MAY ASK YOU TO PROVIDE ADDITIONAL INFORMATION ON SOME FAMILY MEMBERS.

When you are asked to provide information about children, we would like you to include all stillbirths and children who may not have lived past infancy.

Please be as thorough as you can. If you don't know the answer to a question, please write DK in the space provided for the answer. There is space on page 16 for any additional information or other comments you may have.

Please contact the Endocrine Surgery Research Nurse with any questions at [redacted]

**1. On what date was this questionnaire completed?**

|______|______|______|
Month Day Year

**2. Please record your full name, mailing address, and telephone numbers.**

| ______________ | ______________ | ______________ | ________________ |
|---|---|---|---|
| Last Name | First Name | Middle Name | (Maiden Name) |

____________________________________________________________________
Number and Street                                                  Apt. #

| ________________________________ | ______________ | ________________ |
|---|---|---|
| City | State | Zip |

( ______)____________________  ( _____ ) ______________________
Area Code    Home Phone    Area Code    Office Phone

**3. When were you born?**

|______|______|______|
Month Day Year

**4. Where were you born?**

| ________________________________ | __________________ | _____________ |
|---|---|---|
| City | County | State |

OR

| ____________________________________________ | _____________________ |
|---|---|
| Foreign Country | Year moved to U.S.A. |

**5. Are you male or female?**

Male ............................................... ☐
Female ........................................... ☐

**6a. Do you consider yourself to be:**

- Hispanic or Latino ☐
- Not Hispanic or Latino ☐

**6b. What is your race?**

- American Indian or Alaska Native ☐
- Asian ☐
- Black or African American ☐
- Native Hawaiian or Other Pacific Islander ☐
- White ☐

**7. What is your national origin or ancestry? (CHECK NO MORE THAN 4.)**

- England ☐
- Ireland ☐
- Germany ☐
- France ☐
- Italy ☐
- Greece ☐
- Eastern Europe (e.g., Poland, Russia, Hungary, Czechoslovakia) ☐
- Scandinavia (e.g., Norway, Sweden, Denmark, Finland) ☐
- Spain, Portugal ☐
- Other European Countries ☐
- Africa ☐
- Middle East ☐
- India, Pakistan ☐
- China ☐
- Japan ☐
- Other Asian countries or Pacific Islands ☐
- Native American ☐
- Canada ☐
- Mexico ☐
- Puerto Rico ☐
- Central America ☐
- South America ☐
- Other (Specify) ☐

______________________________

**8. Were you raised by someone other than your parents?**

No ☐

Yes: ____________________ ____________________
Name Relationship

____________________ ____________________
Name Relationship

**9.** **Are you a twin or one of a multiple birth?**

No .................................................. ☐ GO TO Q.10
Yes.................................................. ☐ COMPLETE Q.9a-c

**9a.** **How many other infants were born with you?**

________
NUMBER

**9b.** **Of those born with you, how many are of an identical relation with you?**

________
NUMBER

**9c.** **Of those born with you, how many are of a fraternal (non-identical) relation with you?**

________
NUMBER

**10.** **In what religion were you raised? Please note that this religion may differ from the religion that you practice as an adult. (optional)**

None ............................................... ☐
Catholic........................................... ☐
Jewish............................................. ☐
Greek Orthodox ............................... ☐
Mormon........................................... ☐
Seventh Day Adventist..................... ☐
Christian Scientist ............................ ☐
Protestant........................................ ☐
If Protestant, what denomination?
____________________________
Other ............................................... ☐
If Other, please specify
____________________________

**11.** **What is the name, office address, and office phone number of your regular doctor?**

________________ ________________ ________________________
Last Name First Middle

______________________________________________________________
Street Address Apt. #

______________________________ _____________ ______________
City State Zip

( ______ ) ____________________
Area Code Office Phone

**12.** **Did a physician ever state that you had cancer?**

No.................................................. ☐ GO TO Q.13
Yes................................................. ☐ COMPLETE Q.12a-d

**12a.** **What type of cancer did the physician state you had? Where did it begin in your body?**

__________________________ __________________________________
Type Place it Began

**12b.** **When did the physician diagnose this cancer?** ___________
Year

**12c.** **What is the name and office address of the physician who diagnosed this cancer?**

________________ ________________ ________________________
Last Name First Middle

______________________________________________________________
Street Address Apt. #

______________________________ _____________ ______________
City State Zip

**12d. What is the name and address of the hospital or health care facility where the diagnosis was made?**

________________________________________________
Hospital/Clinic Name

________________________________________________
Street Address

| City | State | Zip |
|---|---|---|
| ______________ | ________ | __________ |

**13. What is your marital status?**

- Married or living as married ............. ☐
- Widowed ......................................... ☐
- Separated ....................................... ☐
- Never married ................................. ☐
- Divorced.......................................... ☐
- Remarried ....................................... ☐

IF YOU HAVE EVER BEEN MARRIED, ANSWER Q.14 THROUGH Q.17.
IF YOU PARENTED CHILDREN WITH A PERSON TO WHOM YOU WERE NOT MARRIED, CONSIDER THAT PERSON AS A SPOUSE.

**14. What is the full name and birthdate of your current or most recent spouse?**

| Last Name | First Name | Middle Name | (Maiden Name) |
|---|---|---|---|
| __________ | __________ | __________ | __________ |

Date of Birth |_____|_____|_____|
Month Day Year

**15.** **With this spouse, did you parent any children, including stillbirths and children who may not have lived past infancy?**

Yes.................................................. ☐
No..................................................... ☐

IF THE ANSWER TO Q.15 IS "YES," FILL OUT A GREEN CHART FOR CHILDREN. INSTRUCTIONS FOR FILLING OUT THE CHARTS IN THIS QUESTIONNAIRE APPEAR ON THE YELLOW CHART.

IF YOU HAVE BEEN MARRIED MORE THAN ONCE, ANSWER Q.16 AND Q.17.
IF YOU PARENTED CHILDREN WITH A PERSON TO WHOM YOU WERE NOT MARRIED, CONSIDER THAT PERSON AS A SPOUSE.

IF YOU HAVE NEVER HAD A CHILD, TURN THE PAGE AND COMPLETE THE CHART ON YOUR PARENTS.

**16.** **What is the full name and birthdate of your first spouse?**

| ____________ | _____________ | ____________ | _____________ |
|---|---|---|---|
| Last Name | First Name | Middle Name | (Maiden Name) |

Date of Birth |_____|_____|_____|
Month Day Year

**17.** **Did you parent any children with this spouse?**

Yes.................................................. ☐
No..................................................... ☐

IF THE ANSWER TO Q.17 IS "YES," FILL OUT A SEPARATE GREEN CHART FOR CHILDREN BY THIS FIRST SPOUSE.

IF YOU HAVE BEEN MARRIED MORE THAN TWICE, PLEASE LIST THE NAMES OF YOUR ADDITIONAL SPOUSES AND FILL IN SEPARATE GREEN CHARTS FOR CHILDREN BY ANY ADDITIONAL SPOUSES.

WHEN YOU HAVE COMPLETED ALL CHARTS FOR YOUR CHILDREN, PLEASE TURN TO PAGES 6 AND 7 AND FILL IN THE CHART ON YOUR PARENTS.

**PARENTS'**

| Relationship to you | Name, date of birth and, if living, address and phone number |
|---|---|
| Mother<br><br>☐ ☐ ☐ ☐ ☐ | ______________________________ (__________)<br>Last First Middle Maiden<br><br>______________________________<br>Street Address<br><br>______________________________<br>City State Zip<br><br>Phone: (__________)______________________<br>Area Code Number<br><br>Date of Birth: MO______ DAY______ YR______ |
| Father<br><br>☐ ☐ ☐ ☐ ☐ | ______________________________<br>Last First Middle<br><br>______________________________<br>Street Address<br><br>______________________________<br>City State Zip<br><br>Phone: (__________)______________________<br>Area Code Number<br><br>Date of Birth: MO______ DAY______ YR______ |

**CHART**

<table>
<tr><th rowspan="2">Did this person ever have cancer, a tumor, or a growth?</th><th colspan="2">ANSWER ONLY IF PERSON IS DEAD</th><th rowspan="2">How many children did this couple have?</th></tr>
<tr><th>Date, place of death, cause of death</th><th>Hospital where died</th></tr>
<tr><td>Yes ☐ No ☐ DK ☐<br>________________<br>Type or site Year<br>________________<br>Hospital where diagnosed<br>________________<br>City State</td><td>|_____|_____|_____|<br>Month Day Year<br>________________<br>City<br>________________<br>County State<br>________________<br>Cause</td><td>________________<br>Name of Hospital<br>________________<br>City<br>________________<br>County State<br>________________<br>Physician (if known)</td><td rowspan="2">ENTER THE NUMBER OF CHILDREN YOUR PARENTS HAD, TOGETHER AND WITH OTHER PARTNERS, IN THE SPACE BELOW.<br><br>________<br>Number<br><br>FILL OUT SISTERS & BROTHERS CHART ON THE NEXT PAGE.</td></tr>
<tr><td>Yes ☐ No ☐ DK ☐<br>________________<br>Type or site Year<br>________________<br>Hospital where diagnosed<br>________________<br>City State</td><td>|_____|_____|_____|<br>Month Day Year<br>________________<br>City<br>________________<br>County State<br>________________<br>Cause</td><td>________________<br>Name of Hospital<br>________________<br>City<br>________________<br>County State<br>________________<br>Physician (if known)</td></tr>
</table>

**To whose children does this chart refer?**

## SISTERS' AND BROTHERS'

______________________ | ______________________

**Mother's Name** **Father's Name**

**LIST IN ORDER OF BIRTH**

| Relationship to you | Name, date of birth and, if living, address and phone number |
|---|---|
| Sister ☐<br>Brother ☐<br>Half-Brother (same mother) ☐<br>Half-Brother (same father) ☐<br>Half-Sister (same mother) ☐<br>Half-Sister (same father) ☐<br>☐ ☐ ☐ ☐ ☐ | ______________________ ( ) <br>Last First Middle Maiden<br>______________________<br>Street Address<br>______________________<br>City State Zip<br>Phone: ( ) ______________________<br>Area Code Number<br>Date of Birth: MO______ DAY______ YR______ |
| Sister ☐<br>Brother ☐<br>Half-Brother (same mother) ☐<br>Half-Brother (same father) ☐<br>Half-Sister (same mother) ☐<br>Half-Sister (same father) ☐<br>☐ ☐ ☐ ☐ ☐ | ______________________ ( ) <br>Last First Middle Maiden<br>______________________<br>Street Address<br>______________________<br>City State Zip<br>Phone: ( ) ______________________<br>Area Code Number<br>Date of Birth: MO______ DAY______ YR______ |
| Sister ☐<br>Brother ☐<br>Half-Brother (same mother) ☐<br>Half-Brother (same father) ☐<br>Half-Sister (same mother) ☐<br>Half-Sister (same father) ☐<br>☐ ☐ ☐ ☐ ☐ | ______________________ ( ) <br>Last First Middle Maiden<br>______________________<br>Street Address<br>______________________<br>City State Zip<br>Phone: ( ) ______________________<br>Area Code Number<br>Date of Birth: MO______ DAY______ YR______ |

**CHART**

| Did this person ever have cancer, a tumor, or a growth? | ANSWER ONLY IF PERSON IS DEAD | | How many children did this person have? |
|---|---|---|---|
| | Date, place of death, cause of death | Hospital where died | |
| Yes ☐ No ☐ DK ☐<br>________________<br>Type or site Year<br>________________<br>Hospital where diagnosed<br>________________<br>City State | \|_____\|_____\|_____\|<br>Month Day Year<br>________________<br>City<br>________________<br>County State<br>________________<br>Cause | ________________<br>Name of Hospital<br>________________<br>City<br>________________<br>County State<br>________________<br>Physician (if known) | ________<br>Number<br><br>FILL OUT A WHITE CHART FOR NIECES AND NEPHEWS. |
| Yes ☐ No ☐ DK ☐<br>________________<br>Type or site Year<br>________________<br>Hospital where diagnosed<br>________________<br>City State | \|_____\|_____\|_____\|<br>Month Day Year<br>________________<br>City<br>________________<br>County State<br>________________<br>Cause | ________________<br>Name of Hospital<br>________________<br>City<br>________________<br>County State<br>________________<br>Physician (if known) | ________<br>Number<br><br>FILL OUT A WHITE CHART FOR NIECES AND NEPHEWS. |
| Yes ☐ No ☐ DK ☐<br>________________<br>Type or site Year<br>________________<br>Hospital where diagnosed<br>________________<br>City State | \|_____\|_____\|_____\|<br>Month Day Year<br>________________<br>City<br>________________<br>County State<br>________________<br>Cause | ________________<br>Name of Hospital<br>________________<br>City<br>________________<br>County State<br>________________<br>Physician (if known) | ________<br>Number<br><br>FILL OUT A WHITE CHART FOR NIECES AND NEPHEWS. |

**IF YOU HAVE ADDITIONAL SISTERS AND BROTHERS, FILL OUT THE WHITE SISTERS AND BROTHERS CHART.**

**GRANDPARENTS'**

| Relationship to you | Name, date of birth and, if living, address and phone number |
|---|---|
| Mother's Mother<br>☐ ☐ ☐ ☐ ☐ | ( )<br>Last First Middle Maiden<br>Street Address<br>City State Zip<br>Phone: ( )<br>Area Code Number<br>Date of Birth: MO______ DAY______ YR______ |
| Mother's Father<br>☐ ☐ ☐ ☐ ☐ | Last First Middle<br>Street Address<br>City State Zip<br>Phone: ( )<br>Area Code Number<br>Date of Birth: MO______ DAY______ YR______ |
| Father's Mother<br>☐ ☐ ☐ ☐ ☐ | ( )<br>Last First Middle Maiden<br>Street Address<br>City State Zip<br>Phone: ( )<br>Area Code Number<br>Date of Birth: MO______ DAY______ YR______ |
| Father's Father<br>☐ ☐ ☐ ☐ ☐ | Last First Middle<br>Street Address<br>City State Zip<br>Phone: ( )<br>Area Code Number<br>Date of Birth: MO______ DAY______ YR______ |

**CHART**

| Did this person ever have cancer, a tumor, or a growth? | ANSWER ONLY IF PERSON IS DEAD | | How many children did this person have? |
|---|---|---|---|
| | Date, place of death, cause of death | Hospital where died | |
| Yes ☐ No ☐ DK ☐<br>_______________<br>Type or site Year<br>_______________<br>Hospital where diagnosed<br>_______________<br>City State | \|_____\|_____\|_____\|<br>Month Day Year<br>_______________<br>City<br>_______________<br>County State<br>_______________<br>Cause | _______________<br>Name of Hospital<br>_______________<br>City<br>_______________<br>County State<br>_______________<br>Physician (if known) | ENTER THE NUMBER OF CHILDREN YOUR MOTHER'S PARENTS HAD, TOGETHER AND WITH OTHER PARTNERS, IN THE SPACE BELOW.<br>________<br>Number |
| Yes ☐ No ☐ DK ☐<br>_______________<br>Type or site Year<br>_______________<br>Hospital where diagnosed<br>_______________<br>City State | \|_____\|_____\|_____\|<br>Month Day Year<br>_______________<br>City<br>_______________<br>County State<br>_______________<br>Cause | _______________<br>Name of Hospital<br>_______________<br>City<br>_______________<br>County State<br>_______________<br>Physician (if known) | FILL OUT A PINK AUNTS & UNCLES CHART FOR THESE CHILDREN. |
| Yes ☐ No ☐ DK ☐<br>_______________<br>Type or site Year<br>_______________<br>Hospital where diagnosed<br>_______________<br>City State | \|_____\|_____\|_____\|<br>Month Day Year<br>_______________<br>City<br>_______________<br>County State<br>_______________<br>Cause | _______________<br>Name of Hospital<br>_______________<br>City<br>_______________<br>County State<br>_______________<br>Physician (if known) | ENTER THE NUMBER OF CHILDREN YOUR MOTHER'S PARENTS HAD, TOGETHER AND WITH OTHER PARTNERS, IN THE SPACE BELOW.<br>________<br>Number |
| Yes ☐ No ☐ DK ☐<br>_______________<br>Type or site Year<br>_______________<br>Hospital where diagnosed<br>_______________<br>City State | \|_____\|_____\|_____\|<br>Month Day Year<br>_______________<br>City<br>_______________<br>County State<br>_______________<br>Cause | _______________<br>Name of Hospital<br>_______________<br>City<br>_______________<br>County State<br>_______________<br>Physician (if known) | FILL OUT A PINK AUNTS & UNCLES CHART FOR THESE CHILDREN. |

**PLEASE COMPLETE THIS CHART FOR ANY RELATIVE WITH CANCER FOR WHOM YOU HAVE NOT FILLED OUT INFORMATION IN EARLIER SECTIONS OF THIS QUESTIONNAIRE.**

| Relationship to you | Name, date of birth and, if living, address and phone number |
|---|---|
| ________________ Relationship<br>________________ Mother<br>________________ Father<br>☐ ☐ ☐ ☐ ☐ | ____________________________ ( ) Last First Middle Maiden<br>____________________________ Street Address<br>____________________________ City State Zip<br>Phone: (__________) ________________ Area Code Number<br>Date of Birth: MO______ DAY______ YR______ |
| ________________ Relationship<br>________________ Mother<br>________________ Father<br>☐ ☐ ☐ ☐ ☐ | ____________________________ ( ) Last First Middle Maiden<br>____________________________ Street Address<br>____________________________ City State Zip<br>Phone: (__________) ________________ Area Code Number<br>Date of Birth: MO______ DAY______ YR______ |
| ________________ Relationship<br>________________ Mother<br>________________ Father<br>☐ ☐ ☐ ☐ ☐ | ____________________________ ( ) Last First Middle Maiden<br>____________________________ Street Address<br>____________________________ City State Zip<br>Phone: (__________) ________________ Area Code Number<br>Date of Birth: MO______ DAY______ YR______ |
| ________________ Relationship<br>________________ Mother<br>________________ Father<br>☐ ☐ ☐ ☐ ☐ | ____________________________ ( ) Last First Middle Maiden<br>____________________________ Street Address<br>____________________________ City State Zip<br>Phone: (__________) ________________ Area Code Number<br>Date of Birth: MO______ DAY______ YR______ |

**CHART**

| Did this person ever have cancer, a tumor, or a growth? | ANSWER ONLY IF PERSON IS DEAD | | How many children did this person have? |
|---|---|---|---|
| | Date, place of death, cause of death | Hospital where died | |
| Yes ☐ No ☐ DK ☐<br>__________<br>Type or site (Year)<br>__________<br>Hospital where diagnosed<br>__________<br>City State | \|____\|____\|____\|<br>Month Day Year<br>__________<br>City<br>__________<br>County State<br>__________<br>Cause | __________<br>Name of Hospital<br>__________<br>City<br>__________<br>County State<br>__________<br>Physician (if known) | ______<br>Number |
| Yes ☐ No ☐ DK ☐<br>__________<br>Type or site (Year)<br>__________<br>Hospital where diagnosed<br>__________<br>City State | \|____\|____\|____\|<br>Month Day Year<br>__________<br>City<br>__________<br>County State<br>__________<br>Cause | __________<br>Name of Hospital<br>__________<br>City<br>__________<br>County State<br>__________<br>Physician (if known) | ______<br>Number |
| Yes ☐ No ☐ DK ☐<br>__________<br>Type or site (Year)<br>__________<br>Hospital where diagnosed<br>__________<br>City State | \|____\|____\|____\|<br>Month Day Year<br>__________<br>City<br>__________<br>County State<br>__________<br>Cause | __________<br>Name of Hospital<br>__________<br>City<br>__________<br>County State<br>__________<br>Physician (if known) | ______<br>Number |
| Yes ☐ No ☐ DK ☐<br>__________<br>Type or site (Year)<br>__________<br>Hospital where diagnosed<br>__________<br>City State | \|____\|____\|____\|<br>Month Day Year<br>__________<br>City<br>__________<br>County State<br>__________<br>Cause | __________<br>Name of Hospital<br>__________<br>City<br>__________<br>County State<br>__________<br>Physician (if known) | ______<br>Number |

**IF YOU HAVE ADDITIONAL RELATIVES WITH CANCER, GO TO NEXT PAGE.**

**PLEASE COMPLETE THIS CHART FOR ANY RELATIVE WITH CANCER FOR WHOM YOU HAVE NOT FILLED OUT INFORMATION IN EARLIER SECTIONS OF THIS QUESTIONNAIRE.**

## ADDITIONAL RELATIVES'

| Relationship to you | Name, date of birth and, if living, address and phone number |
|---|---|
| ________________ Relationship<br>________________ Mother<br>________________ Father<br>☐ ☐ ☐ ☐ ☐ | ____________________________ ( ________ )<br>Last First Middle Maiden<br>____________________________<br>Street Address<br>____________________________<br>City State Zip<br>Phone: (__________)____________________<br>Area Code Number<br>Date of Birth: MO______ DAY______ YR______ |
| ________________ Relationship<br>________________ Mother<br>________________ Father<br>☐ ☐ ☐ ☐ ☐ | ____________________________ ( ________ )<br>Last First Middle Maiden<br>____________________________<br>Street Address<br>____________________________<br>City State Zip<br>Phone: (__________)____________________<br>Area Code Number<br>Date of Birth: MO______ DAY______ YR______ |
| ________________ Relationship<br>________________ Mother<br>________________ Father<br>☐ ☐ ☐ ☐ ☐ | ____________________________ ( ________ )<br>Last First Middle Maiden<br>____________________________<br>Street Address<br>____________________________<br>City State Zip<br>Phone: (__________)____________________<br>Area Code Number<br>Date of Birth: MO______ DAY______ YR______ |
| ________________ Relationship<br>________________ Mother<br>________________ Father<br>☐ ☐ ☐ ☐ ☐ | ____________________________ ( ________ )<br>Last First Middle Maiden<br>____________________________<br>Street Address<br>____________________________<br>City State Zip<br>Phone: (__________)____________________<br>Area Code Number<br>Date of Birth: MO______ DAY______ YR______ |

## CHART

| Did this person ever have cancer, a tumor, or a growth? | ANSWER ONLY IF PERSON IS DEAD<br>Date, place of death, cause of death | ANSWER ONLY IF PERSON IS DEAD<br>Hospital where died | How many children did this person have? |
|---|---|---|---|
| Yes ☐ No ☐ DK ☐<br>______ Type or site Year<br>______ Hospital where diagnosed<br>______ City State | \|____\|____\|____\| Month Day Year<br>______ City<br>______ County State<br>______ Cause | ______ Name of Hospital<br>______ City<br>______ County State<br>______ Physician (if known) | ______ Number |
| Yes ☐ No ☐ DK ☐<br>______ Type or site Year<br>______ Hospital where diagnosed<br>______ City State | \|____\|____\|____\| Month Day Year<br>______ City<br>______ County State<br>______ Cause | ______ Name of Hospital<br>______ City<br>______ County State<br>______ Physician (if known) | ______ Number |
| Yes ☐ No ☐ DK ☐<br>______ Type or site Year<br>______ Hospital where diagnosed<br>______ City State | \|____\|____\|____\| Month Day Year<br>______ City<br>______ County State<br>______ Cause | ______ Name of Hospital<br>______ City<br>______ County State<br>______ Physician (if known) | ______ Number |
| Yes ☐ No ☐ DK ☐<br>______ Type or site Year<br>______ Hospital where diagnosed<br>______ City State | \|____\|____\|____\| Month Day Year<br>______ City<br>______ County State<br>______ Cause | ______ Name of Hospital<br>______ City<br>______ County State<br>______ Physician (if known) | ______ Number |

**18. Who else might have more health information about your family?**

NAME: ____________________________________________

ADDRESS: ____________________________________________

____________________________________________

PHONE: (__________) ____________________________________________
AREA CODE NUMBER

RELATIONSHIP TO YOU: ______________________________

NAME: ____________________________________________

ADDRESS: ____________________________________________

____________________________________________

PHONE: (__________) ____________________________________________
AREA CODE NUMBER

RELATIONSHIP TO YOU: ______________________________

---

Please use this space to write in any additional information about your family's medical history or any other comments you may have.

____________________________________________

____________________________________________

____________________________________________

---

**PLEASE BRIEFLY REVIEW THE QUESTIONNAIRE TO MAKE SURE YOU HAVE NOT OMITTED ANY INFORMATION.**

Thank you again for participating in our study of disease in families. Please return all blank charts with your completed questionnaires and charts.

**Appendix 1a - Family History Questionnaire additional pages**

YELLOW

**To whose children does this chart refer?**

**BLUE**

______________________ / ______________________

**Mother's Name** **Father's Name**

## AUNTS' AND UNCLES' CHART

**(Your Father's Sisters and Brothers)**

**LIST IN ORDER OF BIRTH**

| Relationship to you | Name, date of birth and, if living, address and phone number | Did this person ever have cancer, a tumor, or a growth? | ANSWER ONLY IF PERSON IS DEAD | | How many children did this person have? |
|---|---|---|---|---|---|
| | | | Date, place of death, cause of death | Hospital where died | |
| Father's sister ☐<br>Father's brother ☐<br>Father's half-sister ☐<br>Father's half-brother ☐<br>☐☐☐☐☐ | ______ ( ______ )<br>Last First Middle Maiden<br>______<br>Street Address<br>______<br>City State Zip<br>Phone: (______) ______<br>Area Code Number<br>Date of Birth: MO______ DAY______ YR______ | Yes ☐ No ☐ DK ☐<br>______<br>Type or site Year<br>______<br>Hospital where diagnosed<br>______<br>City State | \|_____\|_____\|_____\|<br>Month Day Year<br>______<br>City<br>______<br>County State<br>______<br>Cause | ______<br>Name of Hospital<br>______<br>City<br>______<br>County State<br>______<br>Physician (if known) | ______<br>Number<br>**GO BACK TO QUESTIONNAIRE** |
| Father's sister ☐<br>Father's brother ☐<br>Father's half-sister ☐<br>Father's half-brother ☐<br>☐☐☐☐☐ | ______ ( ______ )<br>Last First Middle Maiden<br>______<br>Street Address<br>______<br>City State Zip<br>Phone: (______) ______<br>Area Code Number<br>Date of Birth: MO______ DAY______ YR______ | Yes ☐ No ☐ DK ☐<br>______<br>Type or site Year<br>______<br>Hospital where diagnosed<br>______<br>City State | \|_____\|_____\|_____\|<br>Month Day Year<br>______<br>City<br>______<br>County State<br>______<br>Cause | ______<br>Name of Hospital<br>______<br>City<br>______<br>County State<br>______<br>Physician (if known) | ______<br>Number<br>**GO BACK TO QUESTIONNAIRE** |
| Father's sister ☐<br>Father's brother ☐<br>Father's half-sister ☐<br>Father's half-brother ☐<br>☐☐☐☐☐ | ______ ( ______ )<br>Last First Middle Maiden<br>______<br>Street Address<br>______<br>City State Zip<br>Phone: (______) ______<br>Area Code Number<br>Date of Birth: MO______ DAY______ YR______ | Yes ☐ No ☐ DK ☐<br>______<br>Type or site Year<br>______<br>Hospital where diagnosed<br>______<br>City State | \|_____\|_____\|_____\|<br>Month Day Year<br>______<br>City<br>______<br>County State<br>______<br>Cause | ______<br>Name of Hospital<br>______<br>City<br>______<br>County State<br>______<br>Physician (if known) | ______<br>Number<br>**GO BACK TO QUESTIONNAIRE** |

**IF YOU NEED SPACE FOR MORE AUNTS AND UNCLES (YOUR FATHER'S SISTERS AND BROTHERS), FILL OUT THE BACK SIDE OF THIS CHART OR START A NEW <u>BLUE</u> AUNTS AND UNCLES CHART. IF YOU HAVE COMPLETED INFORMATION ON ALL OF YOUR FATHER'S SISTERS AND BROTHERS AND THEIR CHILDREN, GO BACK TO PAGE 12 OF THE QUESTIONNAIRE.**

**To whose children does this chart refer?**

______________________ / ______________________

**Mother's Name** **Father's Name**

**BLUE**

## AUNTS' AND UNCLES' CHART

**(Your Father's Sisters and Brothers)**

**LIST IN ORDER OF BIRTH**

| Relationship to you | Name, date of birth and, if living, address and phone number | Did this person ever have cancer, a tumor, or a growth? | ANSWER ONLY IF PERSON IS DEAD: Date, place of death, cause of death | ANSWER ONLY IF PERSON IS DEAD: Hospital where died | How many children did this person have? |
|---|---|---|---|---|---|
| Father's sister ☐<br>Father's brother ☐<br>Father's half-sister ☐<br>Father's half-brother ☐<br>☐☐☐☐☐ | ( ) Last First Middle Maiden<br>Street Address<br>City State Zip<br>Phone: ( ) Area Code Number<br>Date of Birth: MO______ DAY______ YR______ | Yes ☐ No ☐ DK ☐<br>Type or site Year<br>Hospital where diagnosed<br>City State | \|_____\|_____\|_____\| Month Day Year<br>City<br>County State<br>Cause | Name of Hospital<br>City<br>County State<br>Physician (if known) | Number<br>**GO BACK TO QUESTIONNAIRE** |
| Father's sister ☐<br>Father's brother ☐<br>Father's half-sister ☐<br>Father's half-brother ☐<br>☐☐☐☐☐ | ( ) Last First Middle Maiden<br>Street Address<br>City State Zip<br>Phone: ( ) Area Code Number<br>Date of Birth: MO______ DAY______ YR______ | Yes ☐ No ☐ DK ☐<br>Type or site Year<br>Hospital where diagnosed<br>City State | \|_____\|_____\|_____\| Month Day Year<br>City<br>County State<br>Cause | Name of Hospital<br>City<br>County State<br>Physician (if known) | Number<br>**GO BACK TO QUESTIONNAIRE** |
| Father's sister ☐<br>Father's brother ☐<br>Father's half-sister ☐<br>Father's half-brother ☐<br>☐☐☐☐☐ | ( ) Last First Middle Maiden<br>Street Address<br>City State Zip<br>Phone: ( ) Area Code Number<br>Date of Birth: MO______ DAY______ YR______ | Yes ☐ No ☐ DK ☐<br>Type or site Year<br>Hospital where diagnosed<br>City State | \|_____\|_____\|_____\| Month Day Year<br>City<br>County State<br>Cause | Name of Hospital<br>City<br>County State<br>Physician (if known) | Number<br>**GO BACK TO QUESTIONNAIRE** |

**IF YOU NEED SPACE FOR MORE AUNTS AND UNCLES (YOUR FATHER'S SISTERS AND BROTHERS), FILL OUT THE BACK SIDE OF THIS CHART OR START A NEW BLUE AUNTS AND UNCLES CHART. IF YOU HAVE COMPLETED INFORMATION ON ALL OF YOUR FATHER'S SISTERS AND BROTHERS AND THEIR CHILDREN, GO BACK TO PAGE 12 OF THE QUESTIONNAIRE.**

**To whose children does this chart refer?**

PINK

____________________ / ____________________

**Mother's Name** **Father's Name**

## AUNTS' AND UNCLES' CHART

**(Your Mother's Sisters and Brothers)**

**LIST IN ORDER OF BIRTH**

| Relationship to you | Name, date of birth and, if living, address and phone number | Did this person ever have cancer, a tumor, or a growth? | ANSWER ONLY IF PERSON IS DEAD: Date, place of death, cause of death | ANSWER ONLY IF PERSON IS DEAD: Hospital where died | How many children did this person have? |
|---|---|---|---|---|---|
| Mother's sister ☐<br>Mother's brother ☐<br>Mother's half-sister ☐<br>Mother's half-brother ☐<br>☐☐☐☐☐ | ____________ ( _____ )<br>Last First Middle Maiden<br>____________<br>Street Address<br>____________<br>City State Zip<br>Phone: ( ____ ) ________<br>Area Code Number<br>Date of Birth: MO______ DAY______ YR______ | Yes ☐ No ☐ DK ☐<br>____________<br>Type or site Year<br>____________<br>Hospital where diagnosed<br>____________<br>City State | \|_____\|_____\|_____\|<br>Month Day Year<br>____________<br>City<br>____________<br>County State<br>____________<br>Cause | ____________<br>Name of Hospital<br>____________<br>City<br>____________<br>County State<br>____________<br>Physician (if known) | ________<br>Number<br>**GO BACK TO QUESTIONNAIRE** |
| Mother's sister ☐<br>Mother's brother ☐<br>Mother's half-sister ☐<br>Mother's half-brother ☐<br>☐☐☐☐☐ | ____________ ( _____ )<br>Last First Middle Maiden<br>____________<br>Street Address<br>____________<br>City State Zip<br>Phone: ( ____ ) ________<br>Area Code Number<br>Date of Birth: MO______ DAY______ YR______ | Yes ☐ No ☐ DK ☐<br>____________<br>Type or site Year<br>____________<br>Hospital where diagnosed<br>____________<br>City State | \|_____\|_____\|_____\|<br>Month Day Year<br>____________<br>City<br>____________<br>County State<br>____________<br>Cause | ____________<br>Name of Hospital<br>____________<br>City<br>____________<br>County State<br>____________<br>Physician (if known) | ________<br>Number<br>**GO BACK TO QUESTIONNAIRE** |
| Mother's sister ☐<br>Mother's brother ☐<br>Mother's half-sister ☐<br>Mother's half-brother ☐<br>☐☐☐☐☐ | ____________ ( _____ )<br>Last First Middle Maiden<br>____________<br>Street Address<br>____________<br>City State Zip<br>Phone: ( ____ ) ________<br>Area Code Number<br>Date of Birth: MO______ DAY______ YR______ | Yes ☐ No ☐ DK ☐<br>____________<br>Type or site Year<br>____________<br>Hospital where diagnosed<br>____________<br>City State | \|_____\|_____\|_____\|<br>Month Day Year<br>____________<br>City<br>____________<br>County State<br>____________<br>Cause | ____________<br>Name of Hospital<br>____________<br>City<br>____________<br>County State<br>____________<br>Physician (if known) | ________<br>Number<br>**GO BACK TO QUESTIONNAIRE** |

**IF YOU NEED SPACE FOR MORE AUNTS AND UNCLES (YOUR MOTHER'S SISTERS AND BROTHERS), FILL OUT THE BACK SIDE OF THIS CHART OR START A NEW PINK AUNTS AND UNCLES CHART. IF YOU HAVE COMPLETED INFORMATION ON ALL OF YOUR MOTHER'S SISTERS AND BROTHERS, GO BACK TO THE GRANDPARENTS CHART IN THE QUESTIONNAIRE AND SEE OF YOU NEED TO FILL OUT A CHART FOR YOUR MOTHER'S SISTERS AND BROTHERS.**

**To whose children does this chart refer?**

PINK

______________________ / ______________________

**Mother's Name** **Father's Name**

## AUNTS' AND UNCLES' CHART
### (Your Mother's Sisters and Brothers)

**LIST IN ORDER OF BIRTH**

| Relationship to you | Name, date of birth and, if living, address and phone number | Did this person ever have cancer, a tumor, or a growth? | ANSWER ONLY IF PERSON IS DEAD: Date, place of death, cause of death | ANSWER ONLY IF PERSON IS DEAD: Hospital where died | How many children did this person have? |
|---|---|---|---|---|---|
| Mother's sister ☐<br>Mother's brother ☐<br>Mother's half-sister ☐<br>Mother's half-brother ☐<br>☐☐☐☐☐ | ______ ( ______ )<br>Last First Middle Maiden<br>______<br>Street Address<br>______<br>City State Zip<br>Phone: (______) ______<br>Area Code Number<br>Date of Birth: MO______ DAY______ YR______ | Yes ☐ No ☐ DK ☐<br>______<br>Type or site Year<br>______<br>Hospital where diagnosed<br>______<br>City State | \|_____\|_____\|_____\|<br>Month Day Year<br>______<br>City<br>______<br>County State<br>______<br>Cause | ______<br>Name of Hospital<br>______<br>City<br>______<br>County State<br>______<br>Physician (if known) | ______<br>Number<br>**GO BACK TO QUESTIONNAIRE** |
| Mother's sister ☐<br>Mother's brother ☐<br>Mother's half-sister ☐<br>Mother's half-brother ☐<br>☐☐☐☐☐ | ______ ( ______ )<br>Last First Middle Maiden<br>______<br>Street Address<br>______<br>City State Zip<br>Phone: (______) ______<br>Area Code Number<br>Date of Birth: MO______ DAY______ YR______ | Yes ☐ No ☐ DK ☐<br>______<br>Type or site Year<br>______<br>Hospital where diagnosed<br>______<br>City State | \|_____\|_____\|_____\|<br>Month Day Year<br>______<br>City<br>______<br>County State<br>______<br>Cause | ______<br>Name of Hospital<br>______<br>City<br>______<br>County State<br>______<br>Physician (if known) | ______<br>Number<br>**GO BACK TO QUESTIONNAIRE** |
| Mother's sister ☐<br>Mother's brother ☐<br>Mother's half-sister ☐<br>Mother's half-brother ☐<br>☐☐☐☐☐ | ______ ( ______ )<br>Last First Middle Maiden<br>______<br>Street Address<br>______<br>City State Zip<br>Phone: (______) ______<br>Area Code Number<br>Date of Birth: MO______ DAY______ YR______ | Yes ☐ No ☐ DK ☐<br>______<br>Type or site Year<br>______<br>Hospital where diagnosed<br>______<br>City State | \|_____\|_____\|_____\|<br>Month Day Year<br>______<br>City<br>______<br>County State<br>______<br>Cause | ______<br>Name of Hospital<br>______<br>City<br>______<br>County State<br>______<br>Physician (if known) | ______<br>Number<br>**GO BACK TO QUESTIONNAIRE** |

**IF YOU NEED SPACE FOR MORE AUNTS AND UNCLES (YOUR MOTHER'S SISTERS AND BROTHERS), FILL OUT THE BACK SIDE OF THIS CHART OR START A NEW <u>PINK</u> AUNTS AND UNCLES CHART. IF YOU HAVE COMPLETED INFORMATION ON ALL OF YOUR MOTHER'S SISTERS AND BROTHERS, GO BACK TO THE GRANDPARENTS CHART IN THE QUESTIONNAIRE AND SEE OF YOU NEED TO FILL OUT A CHART FOR YOUR MOTHER'S SISTERS AND BROTHERS.**

To whose children does this chart refer?

**GREEN**

______________________ / ______________________
**Mother's Name** **Father's Name**

## CHILDREN'S CHART

**LIST IN ORDER OF BIRTH**

| Relationship to you | Name, date of birth and, if living, address and phone number | Did this person ever have cancer, a tumor, or a growth? | ANSWER ONLY IF PERSON IS DEAD<br>Date, place of death, cause of death | ANSWER ONLY IF PERSON IS DEAD<br>Hospital where died | How many children did this person have? |
|---|---|---|---|---|---|
| daughter ☐<br>son ☐<br>☐☐☐☐☐ | ____________ ( ____ )<br>Last First Middle Maiden<br>____________<br>Street Address<br>____________<br>City State Zip<br>Phone: ( ____ ) ____________<br>Area Code Number<br>Date of Birth: MO______ DAY______ YR______ | Yes ☐ No ☐ DK ☐<br>____________<br>Type or site Year<br>____________<br>Hospital where diagnosed<br>____________<br>City State | \|_____\|_____\|_____\|<br>Month Day Year<br>____________<br>City<br>____________<br>County State<br>____________<br>Cause | ____________<br>Name of Hospital<br>____________<br>City<br>____________<br>County State<br>____________<br>Physician (if known) | ________<br>Number |
| daughter ☐<br>son ☐<br>☐☐☐☐☐ | ____________ ( ____ )<br>Last First Middle Maiden<br>____________<br>Street Address<br>____________<br>City State Zip<br>Phone: ( ____ ) ____________<br>Area Code Number<br>Date of Birth: MO______ DAY______ YR______ | Yes ☐ No ☐ DK ☐<br>____________<br>Type or site Year<br>____________<br>Hospital where diagnosed<br>____________<br>City State | \|_____\|_____\|_____\|<br>Month Day Year<br>____________<br>City<br>____________<br>County State<br>____________<br>Cause | ____________<br>Name of Hospital<br>____________<br>City<br>____________<br>County State<br>____________<br>Physician (if known) | ________<br>Number |
| daughter ☐<br>son ☐<br>☐☐☐☐☐ | ____________ ( ____ )<br>Last First Middle Maiden<br>____________<br>Street Address<br>____________<br>City State Zip<br>Phone: ( ____ ) ____________<br>Area Code Number<br>Date of Birth: MO______ DAY______ YR______ | Yes ☐ No ☐ DK ☐<br>____________<br>Type or site Year<br>____________<br>Hospital where diagnosed<br>____________<br>City State | \|_____\|_____\|_____\|<br>Month Day Year<br>____________<br>City<br>____________<br>County State<br>____________<br>Cause | ____________<br>Name of Hospital<br>____________<br>City<br>____________<br>County State<br>____________<br>Physician (if known) | ________<br>Number |

**IF YOU NEED SPACE FOR MORE CHILDREN, USE THE OTHER SIDE OF THIS CHART OR START A NEW GREEN CHART FOR YOUR CHILDREN. IF YOU HAVE COMPLETED INFORMATION ON ALL OF YOUR CHILDREN WITH ALL OF YOUR SPOUSES, GO BACK TO THE QUESTIONNAIRE AND COMPLETE THE PARENTS' CHART. IF YOU HAVE ANY GRANDCHILDREN WHO HAVE HAD CANCER, FILL OUT INFORMATION ON THESE GRANDCHILDREN ON THE ADDITIONAL RELATIVES CHART AT THE BACK OF THE QUESTIONNAIRE.**

**To whose children does this chart refer?**

**GREEN**

_________________________ / _________________________

**Mother's Name** **Father's Name**

## CHILDREN'S CHART

**LIST IN ORDER OF BIRTH**

| Relationship to you | Name, date of birth and, if living, address and phone number | Did this person ever have cancer, a tumor, or a growth? | ANSWER ONLY IF PERSON IS DEAD<br>Date, place of death, cause of death | Hospital where died | How many children did this person have? |
|---|---|---|---|---|---|
| daughter ☐<br>son ☐<br>☐☐☐☐☐ | ________________ ( ______ )<br>Last First Middle Maiden<br>________________<br>Street Address<br>________________<br>City State Zip<br>Phone: (______) ________<br>Area Code Number<br>Date of Birth: MO______ DAY______ YR______ | Yes ☐ No ☐ DK ☐<br>________<br>Type or site Year<br>________<br>Hospital where diagnosed<br>________<br>City State | \|_____\|_____\|_____\|<br>Month Day Year<br>________<br>City<br>________<br>County State<br>________<br>Cause | ________<br>Name of Hospital<br>________<br>City<br>________<br>County State<br>________<br>Physician (if known) | ________<br>Number |
| daughter ☐<br>son ☐<br>☐☐☐☐☐ | ________________ ( ______ )<br>Last First Middle Maiden<br>________________<br>Street Address<br>________________<br>City State Zip<br>Phone: (______) ________<br>Area Code Number<br>Date of Birth: MO______ DAY______ YR______ | Yes ☐ No ☐ DK ☐<br>________<br>Type or site Year<br>________<br>Hospital where diagnosed<br>________<br>City State | \|_____\|_____\|_____\|<br>Month Day Year<br>________<br>City<br>________<br>County State<br>________<br>Cause | ________<br>Name of Hospital<br>________<br>City<br>________<br>County State<br>________<br>Physician (if known) | ________<br>Number |
| daughter ☐<br>son ☐<br>☐☐☐☐☐ | ________________ ( ______ )<br>Last First Middle Maiden<br>________________<br>Street Address<br>________________<br>City State Zip<br>Phone: (______) ________<br>Area Code Number<br>Date of Birth: MO______ DAY______ YR______ | Yes ☐ No ☐ DK ☐<br>________<br>Type or site Year<br>________<br>Hospital where diagnosed<br>________<br>City State | \|_____\|_____\|_____\|<br>Month Day Year<br>________<br>City<br>________<br>County State<br>________<br>Cause | ________<br>Name of Hospital<br>________<br>City<br>________<br>County State<br>________<br>Physician (if known) | ________<br>Number |

**IF YOU NEED SPACE FOR MORE CHILDREN, USE THE OTHER SIDE OF THIS CHART OR START A NEW GREEN CHART FOR YOUR CHILDREN. IF YOU HAVE COMPLETED INFORMATION ON ALL OF YOUR CHILDREN WITH ALL OF YOUR SPOUSES, GO BACK TO THE QUESTIONNAIRE AND COMPLETE THE PARENTS' CHART. IF YOU HAVE ANY GRANDCHILDREN WHO HAVE HAD CANCER, FILL OUT INFORMATION ON THESE GRANDCHILDREN ON THE ADDITIONAL RELATIVES CHART AT THE BACK OF THE QUESTIONNAIRE.**

To whose children does this chart refer?

____________________ / ____________________

**Mother's Name** **Father's Name**

**WHITE**

**LIST IN ORDER OF BIRTH**

## SISTERS' AND BROTHERS' CHART

| Relationship to you | Name, date of birth and, if living, address and phone number | Did this person ever have cancer, a tumor, or a growth? | ANSWER ONLY IF PERSON IS DEAD | | How many children did this person have? |
|---|---|---|---|---|---|
| | | | Date, place of death, cause of death | Hospital where died | |
| Sister ☐<br>Brother ☐<br>Half-Brother (same mother) ☐<br>Half-Brother (same father) ☐<br>Half-Sister (same mother) ☐<br>Half-Sister (same father) ☐<br>☐ ☐ ☐ ☐ ☐ | ______ ( ____ )<br>Last First Middle Maiden<br>______<br>Street Address<br>______<br>City State Zip<br>Phone: (____) ______<br>Area Code Number<br>Date of Birth: MO______ DAY______ YR______ | Yes ☐ No ☐ DK ☐<br>______<br>Type or site Year<br>______<br>Hospital where diagnosed<br>______<br>City State | \|____\|____\|____\|<br>Month Day Year<br>______<br>City<br>______<br>County State<br>______<br>Cause | ______<br>Name of Hospital<br>______<br>City<br>______<br>County State<br>______<br>Physician (if known) | ______<br>Number<br>FILL OUT A WHITE CHART FOR NIECES AND NEPHEWS. |
| Sister ☐<br>Brother ☐<br>Half-Brother (same mother) ☐<br>Half-Brother (same father) ☐<br>Half-Sister (same mother) ☐<br>Half-Sister (same father) ☐<br>☐ ☐ ☐ ☐ ☐ | ______ ( ____ )<br>Last First Middle Maiden<br>______<br>Street Address<br>______<br>City State Zip<br>Phone: (____) ______<br>Area Code Number<br>Date of Birth: MO______ DAY______ YR______ | Yes ☐ No ☐ DK ☐<br>______<br>Type or site Year<br>______<br>Hospital where diagnosed<br>______<br>City State | \|____\|____\|____\|<br>Month Day Year<br>______<br>City<br>______<br>County State<br>______<br>Cause | ______<br>Name of Hospital<br>______<br>City<br>______<br>County State<br>______<br>Physician (if known) | ______<br>Number<br>FILL OUT A WHITE CHART FOR NIECES AND NEPHEWS. |
| Sister ☐<br>Brother ☐<br>Half-Brother (same mother) ☐<br>Half-Brother (same father) ☐<br>Half-Sister (same mother) ☐<br>Half-Sister (same father) ☐<br>☐ ☐ ☐ ☐ ☐ | ______ ( ____ )<br>Last First Middle Maiden<br>______<br>Street Address<br>______<br>City State Zip<br>Phone: (____) ______<br>Area Code Number<br>Date of Birth: MO______ DAY______ YR______ | Yes ☐ No ☐ DK ☐<br>______<br>Type or site Year<br>______<br>Hospital where diagnosed<br>______<br>City State | \|____\|____\|____\|<br>Month Day Year<br>______<br>City<br>______<br>County State<br>______<br>Cause | ______<br>Name of Hospital<br>______<br>City<br>______<br>County State<br>______<br>Physician (if known) | ______<br>Number<br>FILL OUT A WHITE CHART FOR NIECES AND NEPHEWS. |

**To whose children does this chart refer?**

______________________ / ______________________

**Mother's Name** **Father's Name**

**WHITE**

## SISTERS' AND BROTHERS' CHART

**LIST IN ORDER OF BIRTH**

| Relationship to you | Name, date of birth and, if living, address and phone number | Did this person ever have cancer, a tumor, or a growth? | ANSWER ONLY IF PERSON IS DEAD: Date, place of death, cause of death | ANSWER ONLY IF PERSON IS DEAD: Hospital where died | How many children did this person have? |
|---|---|---|---|---|---|
| Sister ☐<br>Brother ☐<br>Half-Brother (same mother) ☐<br>Half-Brother (same father) ☐<br>Half-Sister (same mother) ☐<br>Half-Sister (same father) ☐<br>☐ ☐ ☐ ☐ ☐ | ( ) Last First Middle Maiden<br>Street Address<br>City State Zip<br>Phone: ( ) Area Code Number<br>Date of Birth: MO______ DAY______ YR______ | Yes ☐ No ☐ DK ☐<br>Type or site Year<br>Hospital where diagnosed<br>City State | \|_____\|_____\|_____\| Month Day Year<br>City<br>County State<br>Cause | Name of Hospital<br>City<br>County State<br>Physician (if known) | Number<br>FILL OUT A <u>WHITE</u> CHART FOR NIECES AND NEPHEWS. |
| Sister ☐<br>Brother ☐<br>Half-Brother (same mother) ☐<br>Half-Brother (same father) ☐<br>Half-Sister (same mother) ☐<br>Half-Sister (same father) ☐<br>☐ ☐ ☐ ☐ ☐ | ( ) Last First Middle Maiden<br>Street Address<br>City State Zip<br>Phone: ( ) Area Code Number<br>Date of Birth: MO______ DAY______ YR______ | Yes ☐ No ☐ DK ☐<br>Type or site Year<br>Hospital where diagnosed<br>City State | \|_____\|_____\|_____\| Month Day Year<br>City<br>County State<br>Cause | Name of Hospital<br>City<br>County State<br>Physician (if known) | Number<br>FILL OUT A <u>WHITE</u> CHART FOR NIECES AND NEPHEWS. |
| Sister ☐<br>Brother ☐<br>Half-Brother (same mother) ☐<br>Half-Brother (same father) ☐<br>Half-Sister (same mother) ☐<br>Half-Sister (same father) ☐<br>☐ ☐ ☐ ☐ ☐ | ( ) Last First Middle Maiden<br>Street Address<br>City State Zip<br>Phone: ( ) Area Code Number<br>Date of Birth: MO______ DAY______ YR______ | Yes ☐ No ☐ DK ☐<br>Type or site Year<br>Hospital where diagnosed<br>City State | \|_____\|_____\|_____\| Month Day Year<br>City<br>County State<br>Cause | Name of Hospital<br>City<br>County State<br>Physician (if known) | Number<br>FILL OUT A <u>WHITE</u> CHART FOR NIECES AND NEPHEWS. |

**To whose children does this chart refer?**

**WHITE**

________________________ / ________________________

**Mother's Name** **Father's Name**

# NIECES' AND NEPHEWS' CHART

**LIST IN ORDER OF BIRTH**

| Relationship to you | Name, date of birth and, if living, address and phone number | Did this person ever have cancer, a tumor, or a growth? | ANSWER ONLY IF PERSON IS DEAD: Date, place of death, cause of death | ANSWER ONLY IF PERSON IS DEAD: Hospital where died | How many children did this person have? |
|---|---|---|---|---|---|
| niece ☐<br>nephew ☐<br>☐☐☐☐☐ | ( ) Last First Middle Maiden<br>Street Address<br>City State Zip<br>Phone: ( ) Area Code Number<br>Date of Birth: MO______ DAY______ YR______ | Yes ☐ No ☐ DK ☐<br>Type or site Year<br>Hospital where diagnosed<br>City State | \|_____\|_____\|_____\| Month Day Year<br>City<br>County State<br>Cause | Name of Hospital<br>City<br>County State<br>Physician (if known) | Number<br>**GO BACK TO QUESTIONNAIRE** |
| niece ☐<br>nephew ☐<br>☐☐☐☐☐ | ( ) Last First Middle Maiden<br>Street Address<br>City State Zip<br>Phone: ( ) Area Code Number<br>Date of Birth: MO______ DAY______ YR______ | Yes ☐ No ☐ DK ☐<br>Type or site Year<br>Hospital where diagnosed<br>City State | \|_____\|_____\|_____\| Month Day Year<br>City<br>County State<br>Cause | Name of Hospital<br>City<br>County State<br>Physician (if known) | Number<br>**GO BACK TO QUESTIONNAIRE** |
| niece ☐<br>nephew ☐<br>☐☐☐☐☐ | ( ) Last First Middle Maiden<br>Street Address<br>City State Zip<br>Phone: ( ) Area Code Number<br>Date of Birth: MO______ DAY______ YR______ | Yes ☐ No ☐ DK ☐<br>Type or site Year<br>Hospital where diagnosed<br>City State | \|_____\|_____\|_____\| Month Day Year<br>City<br>County State<br>Cause | Name of Hospital<br>City<br>County State<br>Physician (if known) | Number<br>**GO BACK TO QUESTIONNAIRE** |

**IF YOU NEED MORE SPACE FOR NIECES AND NEPHEWS, USE THE OTHER SIDE OF THIS CHART OR FILL OUT ANOTHER CHART FOR YOUR NIECES AND NEPHEWS. IF YOU HAVE COMPLETED INFORMATION ON ALL OF YOUR NIECES AND NEPHEWS, GO BACK TO THE QUESTIONNAIRE AND COMPLETE YOUR GRANDPARENTS' CHART.**

To whose children does this chart refer?

**WHITE**

_________________________ / _________________________
**Mother's Name** **Father's Name**

## NIECES' AND NEPHEWS' CHART

**LIST IN ORDER OF BIRTH**

| Relationship to you | Name, date of birth and, if living, address and phone number | Did this person ever have cancer, a tumor, or a growth? | ANSWER ONLY IF PERSON IS DEAD: Date, place of death, cause of death | ANSWER ONLY IF PERSON IS DEAD: Hospital where died | How many children did this person have? |
|---|---|---|---|---|---|
| niece ☐<br>nephew ☐<br>☐☐☐☐☐ | ( ) Last First Middle Maiden<br>Street Address<br>City State Zip<br>Phone: ( ) Area Code Number<br>Date of Birth: MO______ DAY______ YR______ | Yes ☐ No ☐ DK ☐<br>Type or site Year<br>Hospital where diagnosed<br>City State | \|_____\|_____\|_____\| Month Day Year<br>City<br>County State<br>Cause | Name of Hospital<br>City<br>County State<br>Physician (if known) | Number<br>GO BACK TO QUESTIONNAIRE |
| niece ☐<br>nephew ☐<br>☐☐☐☐☐ | ( ) Last First Middle Maiden<br>Street Address<br>City State Zip<br>Phone: ( ) Area Code Number<br>Date of Birth: MO______ DAY______ YR______ | Yes ☐ No ☐ DK ☐<br>Type or site Year<br>Hospital where diagnosed<br>City State | \|_____\|_____\|_____\| Month Day Year<br>City<br>County State<br>Cause | Name of Hospital<br>City<br>County State<br>Physician (if known) | Number<br>GO BACK TO QUESTIONNAIRE |
| niece ☐<br>nephew ☐<br>☐☐☐☐☐ | ( ) Last First Middle Maiden<br>Street Address<br>City State Zip<br>Phone: ( ) Area Code Number<br>Date of Birth: MO______ DAY______ YR______ | Yes ☐ No ☐ DK ☐<br>Type or site Year<br>Hospital where diagnosed<br>City State | \|_____\|_____\|_____\| Month Day Year<br>City<br>County State<br>Cause | Name of Hospital<br>City<br>County State<br>Physician (if known) | Number<br>GO BACK TO QUESTIONNAIRE |

**IF YOU NEED MORE SPACE FOR NIECES AND NEPHEWS, USE THE OTHER SIDE OF THIS CHART OR FILL OUT ANOTHER CHART FOR YOUR NIECES AND NEPHEWS. IF YOU HAVE COMPLETED INFORMATION ON ALL OF YOUR NIECES AND NEPHEWS, GO BACK TO THE QUESTIONNAIRE AND COMPLETE YOUR GRANDPARENTS' CHART.**

## Appendix 2

| Ultrasound feature | Recommendation |
|---|---|
| **Solitary** | |
| microcalcifications/solid/vascular/irregular | FNA if ≥ 1.0 cm |
| adjacent lymphadenopathy | FNA regardless of size |
| complex | FNA if ≥ 1.5 cm |
| Increase in size/close to nerve | Consider FNA |
| **Multiple nodules** | Same criteria per nodule that are ≥ 1.0 cm |

***Criteria for Thyroid nodule FNA biopsy based on American Thyroid Association & Society of Radiologists in Ultrasound Guidelines***
