# Supplementary figures and images for: Co-Occurrence of Familial Non-Medullary Thyroid Cancer (FNMTC) and Hereditary Non-Polyposis Colorectal Cancer (HNPCC) Associated Tumors—A Cohort Study
Source: Front Endocrinol (Lausanne). 2021 Jul 13;12:653401. doi: 10.3389/fendo.2021.653401 (PMC8315151; doi:10.3389/fendo.2021.653401)

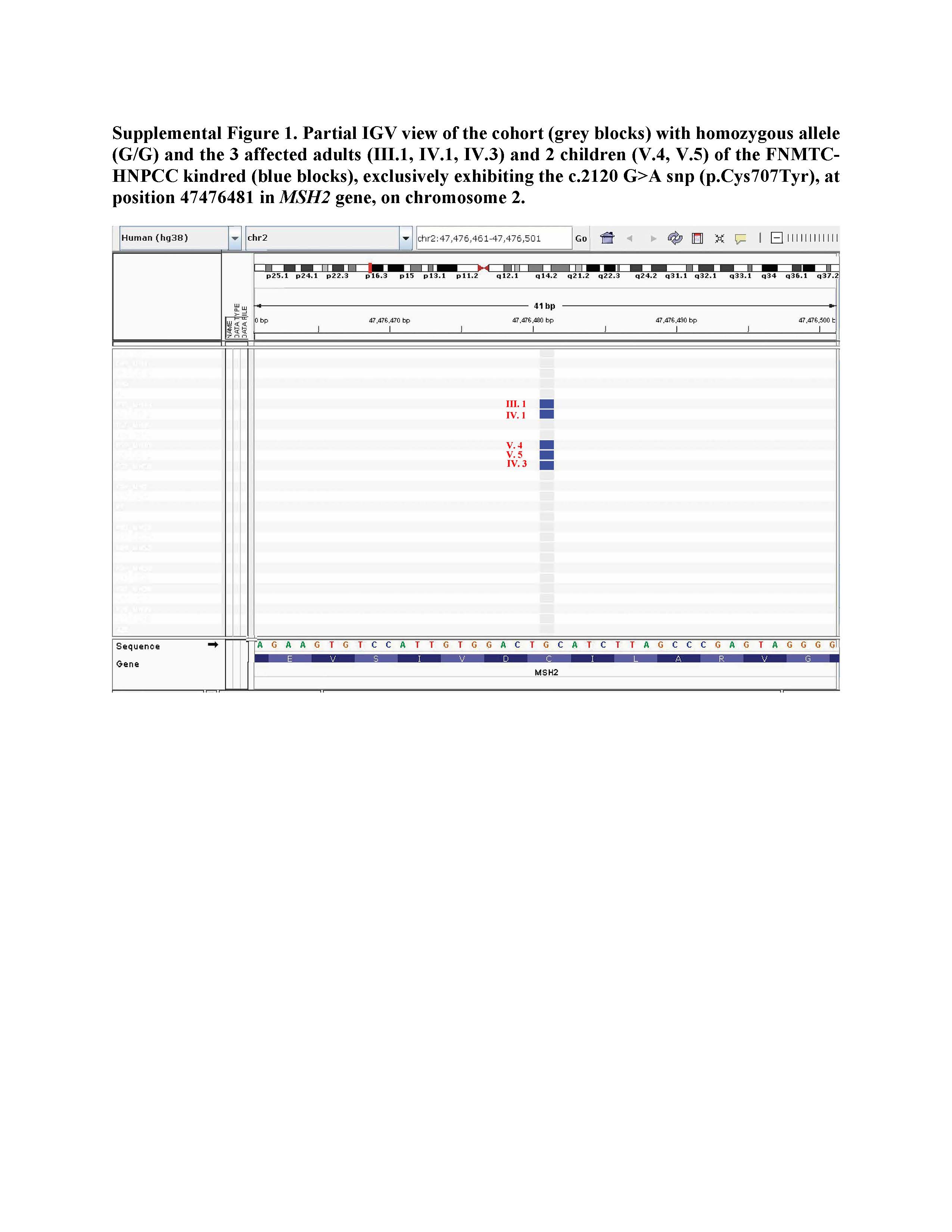

Supplement: Supplementary file 2 [file Image_1.jpeg]

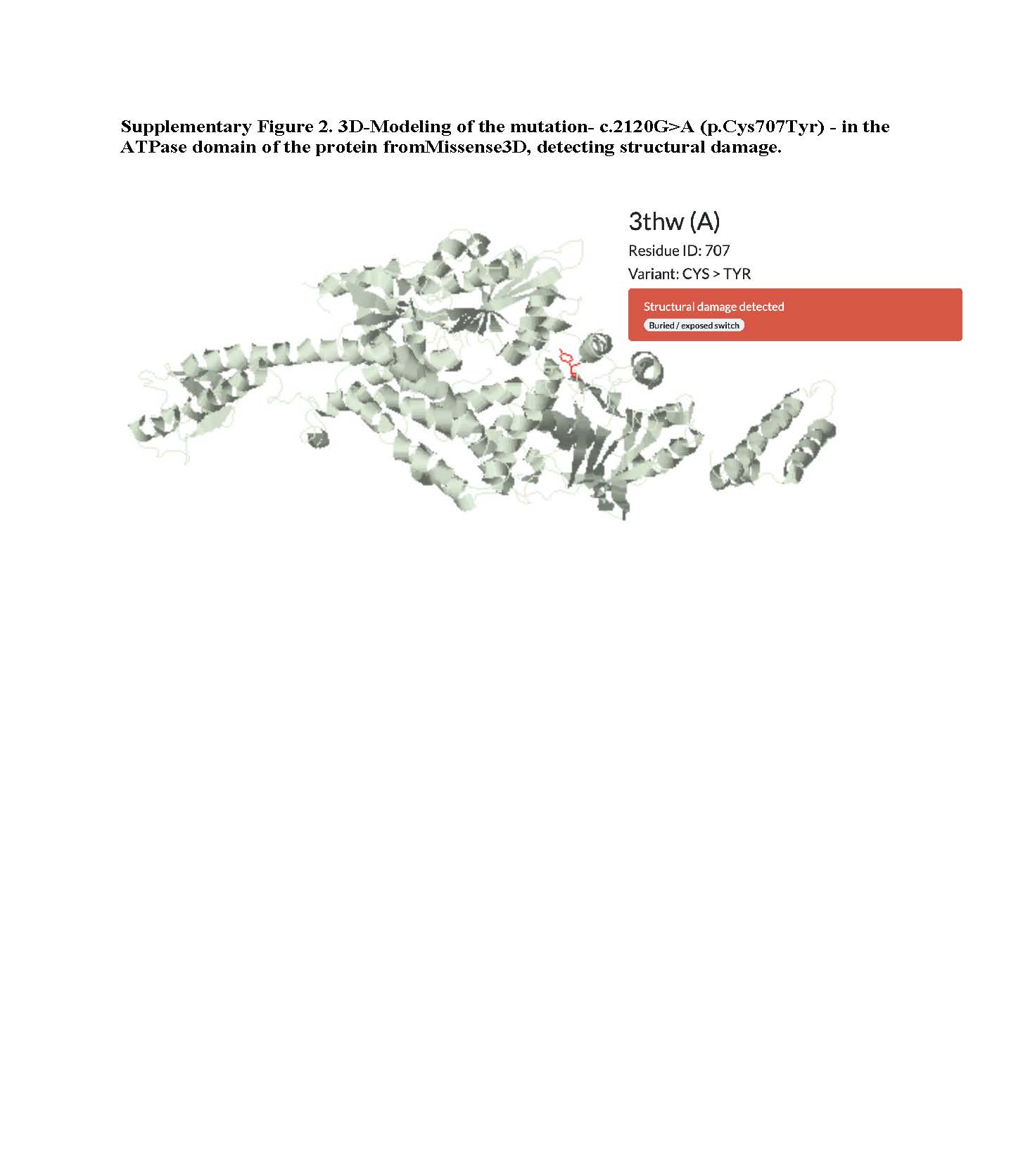

Supplement: Supplementary file 3 [file Image_2.jpeg]
